# Bacterial expression systems based on *Tymovirus*-like particles for the presentation of vaccine antigens

**DOI:** 10.3389/fmicb.2023.1154990

**Published:** 2023-03-23

**Authors:** Anete Ogrina, Ina Balke, Ieva Kalnciema, Dace Skrastina, Juris Jansons, Martin F. Bachmann, Andris Zeltins

**Affiliations:** ^1^Latvian Biomedical Research and Study Centre, Riga, Latvia; ^2^Department of BioMedical Research, University of Bern, Bern, Switzerland

**Keywords:** eggplant mosaic virus, Fel d 1, virus-like particles, *E. coli*, expression

## Abstract

Virus-like particles (VLPs) are virus-derived artificial nanostructures that resemble a native virus-stimulating immune system through highly repetitive surface structures. Improved safety profiles, flexibility in vaccine construction, and the ease of VLP production and purification have highlighted VLPs as attractive candidates for universal vaccine platform generation, although exploration of different types of expression systems for their development is needed. Here, we demonstrate the construction of several simple *Escherichia coli* expression systems for the generation of eggplant mosaic virus (EMV) VLP-derived vaccines. We used different principles of antigen incorporation, including direct fusion of EMV coat protein (CP) with major cat allergen Feld1, coexpression of antigen containing and unmodified (mosaic) EMV CPs, and two coexpression variants of EMV VLPs and antigen using synthetic zipper pair 18/17 (SYNZIP 18/17), and coiled-coil forming peptides E and K (Ecoil/Kcoil). Recombinant Fel d 1 chemically coupled to EMV VLPs was included as control experiments. All EMV-Feld1 variants were expressed in *E. coli*, formed *Tymovirus*-like VLPs, and were used for immunological evaluation in healthy mice. The immunogenicity of these newly developed vaccine candidates demonstrated high titers of Feld1-specific Ab production; however, a comparably high immune response against carrier EMV was also observed. Antibody avidity tests revealed very specific Ab production (more than 50% specificity) for four out of the five vaccine candidates. Native Feld1 recognition and subclass-specific antibody tests suggested that the EMV-SZ18/17-Feld1 complex and chemically coupled EMV-Feld1 vaccines may possess characteristics for further development.

## 1. Introduction

Recent outbreaks of infectious diseases have highlighted that traditional vaccination strategies focusing on attenuated or inactivated viruses may face several limitations, including time-consuming and cost-ineffective manufacturing procedures (Tariq et al., 2022). The widespread coronavirus infectious disease-19 (COVID-19) pandemic has encouraged the development of multiple vaccine candidates and their accelerated progress toward clinical trials, of which mRNA-based technology has shown to be the leading industrial success story. Nevertheless, other technologies for vaccine generation, such as subunit and viral vector vaccines, should not be eliminated in further studies, as their COVID-19 vaccines also provide immunoprotection (Chung et al., 2020).

Immunologically active multivalent structures such as virus-like particles (VLPs) have been extensively studied as new prophylactic and therapeutic vaccine platforms against human or animal infectious agents, cancers and autoimmune diseases (Balke and Zeltins, 2019). VLPs not only have the potential to offer improved safety profiles but can also be manipulated to deliver various antigens and drugs to different targets within tissues and cells by stimulating humoral and cellular immune responses (Nooraei et al., 2021). Furthermore, currently, four VLP-based vaccines against human papilloma virus, malaria and hepatitis B and E viruses are authorized, although many others have entered clinical trials (for review, see Balke and Zeltins, 2019; Nooraei et al., 2021).

A variety of VLPs can be modified to display antigens by using different gene engineering technologies, resulting in recombinant N-, C-, or internal fusions (Frietze et al., 2016). This includes insertion of antigenic sequences onto the VLP surface to achieve optimal epitope presentation without affecting viral morphology (Balke and Zeltins, 2019) since insertions up to 50 aa can easily be tolerated (Lebel et al., 2015) but successful incorporation of proteins of > 300 amino acids in length have been described as well (Walker et al., 2011). A more advanced approach should be considered for placing larger antigens with comparable sizes to the viral coat protein (CP) or antigens with specific biochemical properties. Therefore, mosaic VLPs may overcome these limitations, as they contain unmodified and genetically engineered CPs in the same VLP (Balke and Zeltins, 2019).

Commonly used methods other than molecular cloning are chemical or enzymatic conjugation and physical binding, where foreign antigens are introduced into the structure of VLPs through a specific linkage between two partners (Lebel et al., 2015; Balke and Zeltins, 2019). Chemical coupling of separately purified preassembled carrier VLPs and antigens is achieved by chemical crosslinking agents that modify naturally occurring or engineered surface-exposed lysine residues, creating covalent bonds to the antigen. Several successful examples of vaccines based on chemical coupling against infectious diseases or allergies have been previously published (Zeltins et al., 2017; Gomes et al., 2019; Storni et al., 2019; Rothen et al., 2022). Furthermore, a very efficient enzymatic tool in the vaccine production process based on spontaneous isopeptide bond formation between *Streptococcus pyogenes* bacteria-originated SpyTag/SpyCatcher conjugate partners has recently been described (Ogrina et al., 2022).

Different physical approaches can also be applied in the vaccine construction process for the introduction of peptides or protein domains into VLP structures, which then merge into highly specific, and stable complexes (Balke and Zeltins, 2019). One of many efficient tools that can be used in the construction of vaccines is the artificial complementary coiled-coil-forming oligopeptide pair Ecoil/Kcoil derived from the membrane fusion protein complex SNARE (Litowski and Hodges, 2002; Kumar et al., 2018). The first successful VLP application was heterodimerization using Ecoil/Kcoil demonstrated by Minten et al. (2009) by introducing each binding partner in either CCMV (Cowpea Chlorotic Mottle Virus) or EGFP (enhanced green fluorescent protein), resulting in EGFP-filled capsids. Another option with a similar application was suggested by Reinke et al. (2010) by presenting a set of 23 synthetic coiled-coil pairs (SYNZIPs; SZ). Biophysical characterization of 22 SYNZIP pairs by Thompson et al. (2012) revealed their potential as fusion partners due to the high affinity and specificity between them, which may serve as an attractive protein–protein interaction tool.

Eggplant mosaic virus (EMV) is one of many icosahedral virus species belonging to the *Tymovirus* genus. The members of this genus share many common features, such as size (approximately 30 nm), structural organization, and surface geometry (*T* = 3 symmetry), possession of positive-sense, single-stranded RNA genome, and dicotyledon plants as preferable plant hosts (Martelli et al., 2002). EMV was first reported in infected Solanaceae family plants in Trinidad (Ferguson, 1951), and the complete genome was sequenced by Osorio-Keese et al. (1989) revealing a genome of 6,330 nt residues organized in three open reading frames (ORFs) (Martelli et al., 2002). *Tymovirus* ORF3 codes for the viral *CP* with a size of approximately 20 kDa, which is translated from a subgenomic RNA (sgRNA; 694 nt) (Khan and Dijkstra, 2006).

Several papers highlight the potential of *Tymovirus* VLPs as efficient scaffolds for antigen presentation due to their protein capsid structure position, which includes three chemically identical CP subunits (A, B, and C) that are each located in a different microenvironment and possess a slightly different conformation, such as pentameric capsomeres (A subunits) and hexameric capsomeres (B and C subunits) (Canady et al., 1996; van Roon et al., 2004; Larson et al., 2005; Barnhill et al., 2007). Furthermore, early Turnip yellow mosaic virus (TYMV) studies demonstrated a different type of viral capsid that lacks viral RNA, resulting in the formation of empty shells (“top component”; Matthews, 1960). Later, the same phenomenon was found in other *Tymovirus* members, namely, Physalis mottle virus (PhMV) (Krishna et al., 1999), or Belladonna mottle virus (BDMV) (Savithri et al., 1987). This feature is useful for packaging and drug delivery by producing empty shells *in vitro* (van Roon et al., 2004; Powell and Dreher, 2022). These empty CP shells could serve as drug delivery vehicles (Kim et al., 2018) as they can be loaded with fluorescent dyes (Michels et al., 1999; Barnhill et al., 2007; Masarapu et al., 2017; Hu et al., 2019), chemotherapy drugs (Hu and Steinmetz, 2020), and mammalian cell penetrating enzymes (Kim et al., 2018).

Turnip yellow mosaic virus crystallization experiments by Canady et al. (1996) demonstrated that the N-terminal 26 amino acids of the CP A subunit are disordered, forming flexible chains (Barnhill et al., 2007), and are not essential for VLP self-assembly even after removing the first 26 amino acids of the N-terminus of the CP, resulting in no interference in either particle formation or infectivity (Powell et al., 2012). The same phenomenon was observed for PhMV CP VLPs expressed in *Escherichia coli* (Sastri et al., 1997, 1999) and for Tomato blistering mosaic virus (ToBMV)-like particles in a baculovirus expression system (Vasques et al., 2019), suggesting the high potential of the N-terminal end position as a possible antigen presentation locus. Taking this into account, several authors have demonstrated successful epitope insertion in the N-terminal ends of PhMV CP expressed in *E. coli* cell cultures (Hema et al., 2007; Chandran et al., 2009; Shahana et al., 2015) or model sequence insertions in TYMV CPs using agroinfiltration in *Nicotiana benthamiana* leaves (Shin et al., 2013, 2018), all resulting in the formation of *Tymovirus*-like particles.

Additionally, a study (Barnhill et al., 2007) revealed that approximately 90–120 carboxyl groups of TYMV CP are exposed on the surface and can be used for chemical modifications. Later, studies of PhMV CP C-terminal end deletions demonstrated its significant role in CP folding and capsid assembly, either by removing 5 and 10 amino acids (Sastri et al., 1997), or even in the case of one residue deletion (Sastri et al., 1999), which resulted in lower yield and unstable capsid formation. Furthermore, short peptide (c-Myc, 10 aa) (Shin et al., 2013), or cysteine residue (Shin et al., 2018) insertion in TYMV CP resulted in disruption of VLP assembly. In contrast, opposite results with no interference in capsid assembly were observed when the C-terminal end of TYMV was extended by a 5 aa sequence (Bransom et al., 1995) or a 10 aa poly-histidine tag (Tan et al., 2021), which were the first reports of successfully produced C-terminally modified TYMV chimeric VLPs in *E. coli*.

The main cat allergen Fel d 1 is one of the identified allergens produced by domestic cats (*Felis domesticus*), causing a severe incidence of allergic respiratory diseases worldwide (Bonnet et al., 2018). Fel d 1 has been extensively studied and served as a model antigen in several of our papers as a part of a conjugate vaccine (Zeltins et al., 2017) or in the form of a fusion protein vaccine (Ogrina et al., 2022), inducing strong immune responses. Although Fel d 1 neutralizing Ab production has been detected in Fel d 1-sensitized mice (Zeltins et al., 2017) and cats (Thoms et al., 2020), there is no commercially available vaccine on the market.

**FIGURE 1:**
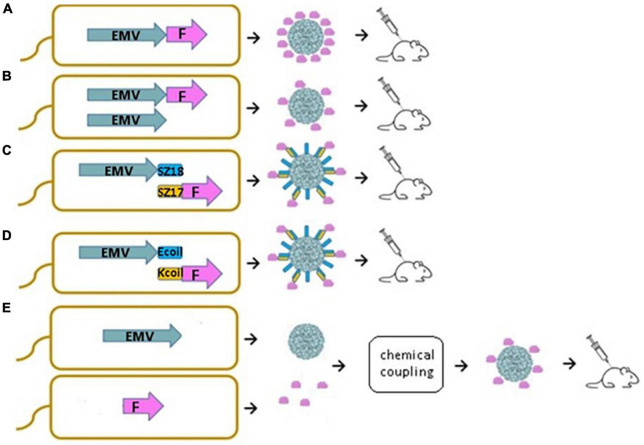
Schematic representation of *E. coli* expression systems used to produce EMV-Feld1 VLPs. **(A)** Direct antigen fusion to the coat protein (CP) of EMV; **(B)** coexpression of antigen-containing EMV CP and unmodified EMV CP for the production of mosaic VLPs; **(C,D)** coexpression of vaccine components allowing the formation of VLP-antigen complexes directly in *E. coli* cells; **(E)** chemical coupling of Feld1 antigen to EMV VLPs. EMV, eggplant mosaic virus; F, Fel d 1 protein; SZ18, synthetic zipper (SZ) 18; SZ17, synthetic zipper (SZ) 17; Ecoil, coiled-coil forming peptide E; Kcoil, coiled-coil forming peptide K.

In this study, we demonstrate that new VLPs derived from EMV CP are suitable for the construction of antigen-containing vaccine candidates. Moreover, we show that antigen sequences can be incorporated into the VLP structure using different genetic, chemical, and physical methods. We introduced a flexible 15 aa linker sequence (GGGGS)_3_ at the C-terminal end of EMV CP by PCR mutagenesis followed by the introduction of the previously described main cat allergen from *F. domesticus* Fel d 1 as a model antigen. We compared four different bacterial expression systems for vaccine generation, such as direct genetic fusion of the antigen with *Tymovirus* EMV CPs (Figure 1A), mosaic VLPs (Figure 1B), and two coexpression variants of EMV VLP and antigen sequences using synthetic zipper pairs 18/17 (SZ18/17) (Figure 1C) and coiled-coil forming peptides E and K (Ecoil/Kcoil) (Figure 1D). All variants were expressed in *E. coli*, formed *Tymovirus*-like VLPs and were used for immunological evaluation in healthy mice individuals. Furthermore, recombinant Fel d 1 (rFeld1) was chemically coupled to EMV CP VLPs and included in mural immunization experiments (Figure 1E) to find the most promising platform for antigen presentation.

## 2. Materials and methods

### 2.1. Cloning of EMV *CP*

The sequence of the EMV *CP* gene (wtEMV; GenBank Accession Number: NC_001480.1) was obtained as a product of gene synthesis (plasmid pUC57-EMV; BioCat, Heidelberg, Germany) with flanking restriction sites *Nde*I and *Xho*I for further subcloning. The wtEMV *CP* gene was excised from the helper plasmid with *Nde*I and *Xho*I restriction enzymes (Thermo Fisher Scientific, Waltham, MA, USA), followed by DNA fragment purification with the GeneJET Gel Extraction Kit (Thermo Fisher Scientific, Waltham, MA, USA) and cloning into the *Nde*I and *Xho*I sites of the *E. coli* expression vector pET42a(+) (Novagen, Madison, WI, USA), resulting in the pET42-EMV expression vector. XL1-Blue Super competent cells (Agilent Technologies, Santa Clara, CA, USA) were used for plasmid preparations. Clones with the corresponding insert were selected by restriction analysis by digestion with *Nde*I and *Xho*I.

### 2.2. Construction of the EMV direct fusion and mosaic system with Feld1

The *Fel d 1* gene used in this study has been described previously (Zeltins et al., 2017; Ogrina et al., 2022). For the construction of the EMV-Feld1 direct fusion and mosaic system, we introduced *Bam*HI and *Xho*I sites in the Fel d 1 gene by PCR mutagenesis using the primers Feld_BamF (5′ CAG GAT CCG AAA TTT GTC CGG CAG TTA AAC G 3′) and Feld_XhoR (5′ CTC GAG TTA TTA ACG ACC CAG GGT ATT CAG TTT C 3′) and Taq DNA polymerase (Thermo Fisher Scientific, Waltham, MA, USA). PCRs were carried out in a 96-well thermal cycler Veriti (Thermo Fisher Scientific, Waltham, MA, USA). The PCR product was subcloned into the linearized vector pTZ-57 using the InsTAclon PCR Cloning Kit (Thermo Fisher Scientific, Waltham, MA, USA), resulting in pTZ-Feld1-Bam-Xho. PCR fragment-containing clones were selected by digestion analysis using *Bam*HI and *Xho*I restriction enzymes (Thermo Fisher Scientific, Waltham, MA, USA). For all PCR-derived plasmids, three positive clones were sequenced by the Sanger sequencing method using the ABI PRISM BigDye Terminator v3.1 Ready Reaction Cycle Sequencing Kit (Thermo Fisher Scientific, Waltham, MA, USA) and ABI PRISM 3130xl sequencer (Thermo Fisher Scientific, Waltham, MA, USA) with the corresponding primers M13seq-F (5′ GCC AGG GTT TTC CCA GTC ACG A 3′) and/or M13seq-R (5′ GAG CGG ATA ACA ATT TCA CAC AGG 3′). The clone without PCR errors was used for further development of the Fel d 1 expression vector.

For the EMV *CP* gene, we introduced a flexible linker (GGGGS)_3_ and *Bam*HI site coding sequences at the 3′-terminus. The EMV CP 3′-terminal fragment and corresponding additional sequences were amplified in PCR mutagenesis using long overlapping primers EMV-g4sF (5′ CCA TGG CAA GCT CCG CTT GTC CTC CCC CCT CCT CCA AGC CAA TGG AGG AGG TGG TAG TGG TGG AGG TGG ATC T 3′) and EMV-g4sR (5′ CTC GAG AAG CTT ATT AGG ATC CAC CTC CTC CAG ATC CAC CTC CAC CAC TAC CAC CTC CTC C 3′) without template. A PCR fragment (3′EMV-CG4S) containing clones (pTZ-3′EMV-CG4S) was selected after sequencing several plasmid clones. The *Nco*I/*Xho*I fragment from pTZ-3′EMV-CG4S was further ligated into the pET42-EMV vector digested with the same enzymes, resulting in the pET42-EMV-CG4S vector. The corresponding vector clone was selected by digestion analysis using *Nde*I/*Xho*I restriction enzymes.

The *Fel d 1* sequence was excised from pTZ-Feld1-Bam-Xho with *Bam*HI and *Xho*I restriction enzymes and ligated into the linearized vector pET42-EMV-CG4S at the same sites. The correct clone of the direct fusion plasmid pET42-EMV-CG4S-Feld1 was selected by digestion analysis with *Sty*I (Thermo Fisher Scientific, Waltham, MA, USA) and *Xho*I.

For the mosaic system, we introduced flanking *Psc*I and *Sal*I restriction enzyme sites into the EMV CP gene by PCR mutagenesis using the following primers: EMV-PscF (5′ ACA TGT CTG AAG ACA CAG CAA TCA TCA GAA 3′) and EMV-SalR (5′ GTC GAC TTA TTA ATT GGC TTG GAG GAG GGG GGA 3′). The pTZ-57 plasmid containing the PCR fragment (EMV-Psc) was selected by digestion analysis using *Psc*I and *Sal*I restriction enzymes (Thermo Fisher Scientific, Waltham, MA, USA). After verification by Sanger sequencing, the corresponding EMV-Psc fragment was excised from pTZ-EMV-Psc by *Psc*I and *Sal*I restriction enzymes (Thermo Fisher Scientific, Waltham, MA, USA) and ligated into the pETDuet-1 (Novagen, Madison, WI, USA) vector under the first T7 promoter in the cloning sites *Nco*I (Thermo Fisher Scientific, Waltham, MA, USA) and *Sal*I. A correct clone of the resulting pETDu-EMV was found after restriction analysis using the *Not*I enzyme.

The plasmid pETDu-EMV was further used for subcloning of the EMV-CG4S-Feld1 gene from the pET42-EMV-CG4S-Feld1 vector under the second T7 promoter using *Nde*I and *Xho*I digestion enzymes. The plasmid clone encoding proteins in the mosaic system (pETDu-mEMV-CG4S-Feld1) was selected by digestion analysis with *Nco*I and *Xho*I.

### 2.3. Cloning of complementary coiled-coil forming peptides *K* and *E*

The coding sequences of coiled-coil peptides E (Ecoil) and K (Kcoil) were obtained from overlapping oligonucleotides in PCRs without a template. The Ecoil coding sequence was amplified using primers Ecoil-BamF (5′ CAG GAT CCG AAA TTG CTG CTC TCG AAA AAG AGA TCG CGG CGC TCG AAA AAG AAA TT 3′) and Ecoil-XhoR (5′ CTC GAG AAG CTT ATT TTT CAA GAG CAG CAA TTT CTT TTT CGA GCG CCG CGA 3′). The *Ecoil* coding sequence was introduced after the (GGGGS)_3_ linker-coding sequence into the vector pET42-EMV-CG4S using cloning sites *Bam*HI and *Xho*I.

For the Kcoil expression vector, the Kcoil coding sequence was also obtained by PCR using the following oligonucleotides: Kcoil-NcoF (5′ CCA TGG GAA AGA TCG CGG CCC TCA AAG AAA AGA TCG CCG CCC T 3′) and Kcoil-BamR (5′ GGA TCC TTC CTT CAG GGC CGC AAT CTT TTC CTT GAG GGC GGC GAT CTT TTC T 3′). The PCR product was cloned into the pTZ57 vector, and after verifying the sequence, it was introduced into the pACYCDuet-1 vector at the *Nco*I and *Bam*HI sites. Next, the Feld1 sequence was introduced into the resulting vector pACYCDu-Kcoil(3x) between the cloning sites *Bam*HI and *Xho*I. Both Ecoil- and Kcoil-containing plasmids were sequenced and used for coexpression of EMV-CG4S-Ecoil(3x) and Kcoil(3x)-Feld1 proteins.

### 2.4. Cloning of synthetic coiled-coil anti-parallel zipper pair SYNZIP 17/18

The corresponding sequences for the synthetic zipper pair *SYNZIP (SZ) 17* and *18* were obtained from published Supplementary material by Reinke et al. (2010) and purchased as a gene synthesis product in plasmid pUC57 (BioCat, Germany). For further subcloning, the plasmid pUC57-SZ17 (Amp) contained the introduced restriction sites of *Nco*I and *Hin*dIII, and pUC57-SZ18 (Amp) contained the *Nde*I and *Xho*I restriction sites. For convenient protein coexpression in *E. coli* cells, each SZ pair was subcloned into vector plasmids with different antibiotic resistances. SZ17 was subcloned into the commercial vector plasmid pRSFDuet-1 (Novagen, USA) with the kanamycin resistance gene using previously introduced *Nde*I*/Xho*I restriction sites, and SZ18 was subcloned into pET-Duet1 (Novagen, USA) with the ampicillin resistance gene using *Nco*I*/Hin*dIII sites, resulting in plasmids pRSF-Duet-SZ17 and pET-Duet-SZ18.

For the Feld1 antigen-SYNZIP 17 fusion, the pTZ-Feld1-Bam-Xho plasmid was digested with *Bam*HI*/Xho*I and subcloned into the same sites of pRSF-Duet-SZ17 after partial restriction enzyme treatment, resulting in the expression vector pRSF-SZ17-Feld1. Additionally, for convenient protein purification, the 6xHis-tag coding sequence was introduced into the constructed pRSF-SZ17-Feld1 sequence by subcloning the *Msc*I*/Xho*I fragment from plasmid pET42-Feld1-C6H (Zeltins et al., 2017) into pRSF-SZ17-Feld1 digested with the same enzymes. The sequence of the resulting plasmid pRSF-SZ17-Feld1-C6H was verified by sequencing to confirm the introduction of His-tag coding DNA.

To obtain the EMV *CP*-*SYNZIP 18* fusion construct, the previously mentioned EMV *CP*-containing plasmid for direct fusion pET42-EMV-CG4S was digested with *Bam*HI*/Sph*I, and subcloned into the pET-Duet-SZ18 plasmid cut by the same restriction enzymes, resulting in the expression vector pETDu-EMV-CG4S-SZ18. Several plasmid clones were sequenced to confirm the introduction of the SZ18-EMV sequence.

### 2.5. Expression and purification of EMV CP VLPs and EMV-Feld1-containing VLPs

First, all constructed expression vectors coding for wtEMV CP, direct fusion (EMV-CG4S-Feld1), and mosaic fusion (mEMV-CG4S-Feld1) were transformed into *E. coli* C2566 cells (New England, USA). For coiled-coil partners, two expression strategies were used. First, EMV-CG4S-SZ18- and EMV-CG4S-Ecoil-containing plasmids as well as plasmids coding for Feld1 expression SZ17-Feld1-C6H and Kcoil-Feld1 were expressed separately by directly transforming them into *E. coli* C2566 cells (New England, USA). Second, coexpression of EMV-Ecoil/Kcoil-Feld1 and EMV-SZ18/SZ17-Feld1 plasmids in *E. coli* C2566 cells was performed. After expression, the highest yield producing clones for the target protein were cultivated in 2TY medium (1,6% tryptone, 1,0% yeast extract, and 0.5% NaCl) with the addition of the corresponding antibiotic (kanamycin for pET42 and pRSF-derivatives, 25 mg/L; ampicillin for pETDuet-1 derivatives, 100 mg/L; and chloramphenicol for pACYCDuet-1 derivatives, 25 mg/L). Cultivation conditions were as previously described (Balke et al., 2018). In brief, flasks with cells were cultivated using an Ecotron HT table-top incubation shaker (200 rev/min; Infors, Switzerland) at 30°C until the OD reached 0.8–1.0. After induction with 0.2 mM isopropyl β-d-1-thiogalactopyranoside (IPTG), cells were further cultivated at 20°C for 18 h, collected by low-speed centrifugation (8,228 × *g*, 5 min, 5°C, Eppendorf 5804R, Germany) and kept frozen at −70°C until use.

The cells containing wtEMV CP were thawed on ice, suspended in 1× PBS and disrupted using an ultrasonic device (Hielscher 200, power 70%, pulse 50%, 16 min) on ice. The same procedure was applied for direct fusion and mosaic EMV-Feld1 using PBS buffer containing 5 mM β-mercaptoethanol (β-ME). For separately expressed EMV-CG4S-Ecoil, EMV-CG4S-SZ18, Kcoil-Feld1, and SZ17-Feld1-C6H for both coexpression pairs EMV-Ecoil/Kcoil-Feld1 and EMV-SZ18/SZ17-Feld1, 1× PBS with an additional 5 mM β-ME and 0.5% TX-100 was used in the cell disruption procedure with the same parameters. The solutions were collected, and cell debris was removed by centrifugation (15,557 × *g*, 10 min, 5°C).

The purification was performed by ultracentrifugation for all constructs except for wtEMV VLPs and SZ17-Feld1-C6H in a sucrose gradient (20–60% sucrose in 1× PBS with additional 0.5% TX-100) as described in our previous paper (Ogrina et al., 2022). After ultracentrifugation, fractions were collected and analyzed by SDS-PAGE to select fractions with EMV-Feld1-containing VLPs or Kcoil-Feld1 protein. VLP samples were then combined and transferred to a new ultracentrifugation tube followed by a second round of ultracentrifugation to sediment the VLPs (Optima L-100XP; Type70Ti rotor, Beckman, USA; 183,960 × *g*, 4 h, 4°C). The pellet was then dissolved in 1× PBS. Soluble wtEMV proteins were purified by polyethylene glycol (PEG) precipitation by adding 8% PEG8000 and 0.3 M NaCl to the cell lysate. After incubation, the VLP precipitate was separated from cellular proteins (15,557 × *g*, 10 min, 5°C), and the pellet was dissolved in 1× PBS. Precipitation was repeated three more times.

After purification or precipitation, all EMV CP VLPs (wtEMV; EMV-Feld1, mEMV-Feld1, EMV-CG4S-Ecoil; EMV-CG4S-SZ18, EMV-SZ18/SZ17-Feld1 and EMV-Ecoil/Kcoil-Feld1) were further purified using ultracentrifugation through the 30% sucrose “cushion” in 1× PBS supplemented with 0.5% TX-100 (Optima L-100XP; Type70Ti rotor, Beckman, USA; 183,960 × *g*, 4 h, 4°C). Pellets were then dissolved in 1× PBS and, if necessary, concentrated using an Amicon Ultra-15, 100 K filtration unit (Merck-Millipore, USA), keeping the final VLP products stored at 4°C.

Kcoil-Feld1 protein after purification in a sucrose gradient (Supplementary Figure 10) was dialyzed against 100 volumes of 1× PBS followed by purification with gel filtration on a ÄKTA Pure 25 XK 16/70 Superdex200 column (GE Healthcare, USA). Purified proteins were eluted with 1× PBS at 1 ml/min, collecting 2 ml per fraction. Separately expressed SZ17-Feld1-C6H was purified on a His-tag column and dialyzed as previously described (Zeltins et al., 2017).

The concentrations of all purified proteins were estimated using a Qubit Protein Assay Kit (Thermo Fisher Scientific, USA) and Qubit 2.0 fluorometer (Thermo Fisher Scientific, USA) according to the manufacturer’s recommendations. All steps in the purification/precipitation process were monitored by using 12.5% SDS-PAGE and agarose gels and Western blot (WB) analysis.

### 2.6. Chemical coupling of wtEMV CP VLPs and rFeld1

rFel d 1 was expressed and purified as previously described (Zeltins et al., 2017). For chemical coupling, the crosslinker succinimidyl-6-(β-maleimidopropionamido)-hexanoate (SMPH; Thermo Fisher Scientific, USA) was used for rFel d 1 conjugation to wtEMV CP VLPs. A threefold molar excess of SMPH to EMV VLPs was used for the reaction at 23°C for 1 h. Unreacted SMPH was removed by an Amicon Ultra-15, 50 K centrifugal filter (Merck-Millipore, USA) washing with 1× PBS four times (4 × 6 min) at 3,214 × *g* (5,000 rpm) and 5°C. The antigen was treated with a 10-fold molar excess of the mild reducing agent tris (2-carboxyethyl) phosphine (TCEP; Sigma-Aldrich, Burlington, MA, USA) for 10 min at 23°C prior to chemical conjugation. The coupling was performed by adding a twofold molar excess of rFel d 1 to the SMPH-derivatized wtEMV VLPs at 23°C for 2 h by shaking at 1,400 rpm/min on a DSG Titertek (Flow Laboratories, Oldham, UK). Free rFel d 1 was removed using an Amicon Ultra-15, 50 K centrifugal filter (Merck-Millipore, USA) by washing with 1× PBS four times (4 × 6 min) at 3,214 × *g* (5,000 rpm) and 5°C. All stages of coupling were monitored by SDS-PAGE and native agarose gel, and the integrity of VLPs was confirmed by transmission electron microscopy (TEM).

### 2.7. Binding of coiled-coil partners *in vitro*

After purification, separately expressed coiled-coil pairs EMV-CG4S-Ecoil and Kcoil-Feld1 or EMV-CG4S-SZ18 and SZ17-Feld1-C6H were mixed together in a 1:1 ratio according to their concentrations in a volume of 100 μl and incubated for 1 h at room temperature (RT). Additionally, as EMV-SZ18/SZ17-Feld1-C6H contains a 6-histidine tag, it was purified using a His-tag column as previously described and dialyzed against 100 volumes of 1× PBS followed by 12.5% SDS-PAGE, NAG analysis and TEM. All steps from the binding process were analyzed in SDS-PAGE and agarose gels and examined under TEM as well (Supplementary Figures 9, 11). Concentrations of protein samples were measured by a Qubit 2.0 fluorometer (Thermo Fisher Scientific, USA) with a Qubit Protein Assay Kit (Thermo Fisher Scientific, USA) according to the manufacturer’s recommendations.

### 2.8. Transmission electron microscopy

All steps for purified EMV CP VLP visualization in TEM were performed as previously described in our paper (Ogrina et al., 2022). At least five pictures were captured per sample.

### 2.9. Immunological evaluation of EMV CP-Feld1-containing VLPs

#### 2.9.1. Western blot analysis

Western blot analysis was performed as described in Ogrina et al. (2022). First, samples for EMV-Feld1-containing VLPs were loaded and run on 17-well 1.0 mm Bolt 4–12% Bis-Tris Plus gel (Thermo Fisher Scientific, USA). The corresponding proteins were then transferred onto an Amersham Protran 0.45 μm nitrocellulose membrane (GE Healthcare, Piscataway, USA) for antibody detection using a semidry blotting apparatus (45 mA, 45 min). A membrane was then blocked with 1% alkali-soluble casein (Merck-Millipore, USA) for 1 h at RT followed by incubation overnight at 4°C in anti-EMV and anti-Feld1 polyclonal antibodies (Ab’s) (dilution 1: 1,000 in PBS with 1% alkali-soluble casein, in-house produced). After a 15 min washing step with TBS buffer (150 mM NaCl; 10 mM Tris pH 7.5), membranes were incubated for 3 h with horseradish peroxidase-conjugated anti-mouse IgG (Sigma-Aldrich, USA; 1:1,000 in PBS with 1% alkali-soluble casein) at RT. After a repeated washing step with TBS buffer, signals were detected by incubating the membrane in TBS buffer supplemented with peroxidase substrates (0.002% *o –* dianisidine and 0.03% hydrogen peroxide).

#### 2.9.2. Vaccination

Female BALB/c mice (5 per group, 6–8 weeks, Laboratory Animal Centre, University of Tartu, Estonia) were vaccinated subcutaneously (s.c.) with 30 μg of total protein of each VLP (five groups – direct, mosaic, Ecoil/Kcoil, SZ18/SZ17, and chemically coupled) in 300 μl of PBS on Day 0. Mice were boosted on Days 14 and 28 with the same dosage of VLPs. Serum samples from each mouse were collected on Days 0, 14, and 28, and final bleeding was performed on Day 42. All animal-related procedures in this study were approved by the Animal Protection Ethical Committee of the Latvian Food and Veterinary Service (permission No. 89).

#### 2.9.3. Enzyme-linked immunosorbent assay

Enzyme-linked immunosorbent assay (ELISA) tests were performed as described in Ogrina et al. (2022). The plates (96-well; Nunc Immuno MaxiSorp, Rochester, NY, USA, Thermo Fisher Scientific, USA) were coated with 100 μl of 10 μg/ml purified samples (EMV CP or rFeld1) in 50 mM sodium carbonate buffer (pH 9.6) overnight at 4°C. After three washes of the plates with PBS-0.05% Tween-20 solution and blocking with 1% BSA in PBS at 37°C for 1 h, 100 μl of serially diluted mouse sera were added to the wells and incubated for 1 h at 37°C. Then, the washing step was repeated three times, followed by incubation with 100 μl of secondary antibodies (anti-mouse IgG conjugated to horseradish peroxidase, dilution 1: 10,000; Sigma-Aldrich, USA; 1 h, 37°C). Finally, plates were washed once again three times, and the substrate (o-phenylenediamine dihydrochloride, OPD; Sigma-Aldrich, USA) was added for color development. The absorbance measurements were performed using a Labsystems 353 Multiscan MS microplate reader (Sweden) at 492 nm. Final titers were calculated as the highest absorbance values that exceeded threefold of the negative control (non-immunized mouse serum) which was collected from all five immunization groups on Day 0 and pooled (5 μl from each mouse per group).

Monoclonal Feld1 antibody ELISA tests were similarly performed by replacing the immunized mouse sera with two commercially available Feld1 mAbs as described previously according to manufacturers’ guidelines (MA-3E4 and MA-6F9, Indoor Biotechnologies, UK; Ogrina et al., 2022). After blocking with 1% BSA in PBS at 37°C for 1 h, serial dilutions of two types of mAbs were added to plates and incubated for 1 h 37°C and then washed three times with PBS containing 0.05% Tween-20. The rest of the procedure was the same as to that described above. Non-immunized mouse serum was used as a negative control and titer calculations were performed as described above.

To assess the subclass antibody response (IgG1 and IgG2a), isotype-specific ELISA experiments were performed for both anti-EMV and anti-Feld1 Ab detection for each mouse from sera obtained from Day 42 following the manufacturer’s protocol (mouse mAb antibody isotyping reagent ISO2-1KT kit; Sigma-Aldrich, USA). For secondary Abs, a peroxidase conjugate of monoclonal anti-goat/sheep IgG Abs (Sigma-Aldrich, USA) was used, and endpoint titers were calculated as previously described.

#### 2.9.4. Avidity ELISA

Avidity ELISA is performed as previously described ELISA test with one additional washing step and was performed following the protocol described in our previous papers (Mohsen et al., 2021; Ogrina et al., 2022) using sera obtained on Day 42 from each mouse: two sets of plates were coated with 10 μg/ml EMV- or rFeld1-derived proteins. After serum incubation plates were washed separately, one set of plates was washed three times with 50 μl/well of a 7 M urea solution in PBS-0.05% Tween-20 for 5 min, whereas other plates were washed with the same volume of PBS-0.05% Tween-20. For the rest of the washing steps, a solution of PBS-0.01% Tween-20 was used. The following procedure was identical to that reported above.

#### 2.9.5. ELISA for native Feld1

For native Feld1 (nFeld1) – EMV-Feld1 containing VLP variants, all five constructed VLP variants at a concentration of 10 μg/ml in 50 mM sodium carbonate buffer (pH 9.6, 100 μl per well) were coated on ELISA plates and stored at 4°C ON. Final mouse serum samples (5 mice per group) from each of five immunization groups were pooled (5 μl from each mouse), serially diluted, added to the wells, and incubated as previously described. The rest of the ELISA test was performed as described before. The endpoint titers were calculated as stated above.

## 3. Results

### 3.1. EMV CP Feld1 VLP construction and characterization

Our previous results demonstrated that a flexible 15 aa long glycine-serine linker sequence [(GGGGS)_3;_ G4S] improved the overall flexibility of the Feld1 antigen dimer (Zeltins et al., 2017) and allowed the folding and VLP assembly of potato virus Y (PVY) CP-antigen fusion (Kalnciema et al., 2012; Ogrina et al., 2022). The usefulness of the G4S linker was previously shown for C-terminal fusions to Tobacco mosaic virus (TMV) CP (Werner et al., 2006). Several prior failed attempts with *Tymovirus* CP C-terminal fusions have been noted (Shin et al., 2013, 2018); therefore, we assumed that adding the G4S linker sequence should facilitate VLP formation after genetic fusion of foreign antigens. We constructed the C-terminally extended EMV CP resulting in EMV-CG4S, which formed intact icosahedral VLPs with a size of ∼28–30 nm after expression in *E. coli* and purification (data not shown).

**FIGURE 2:**
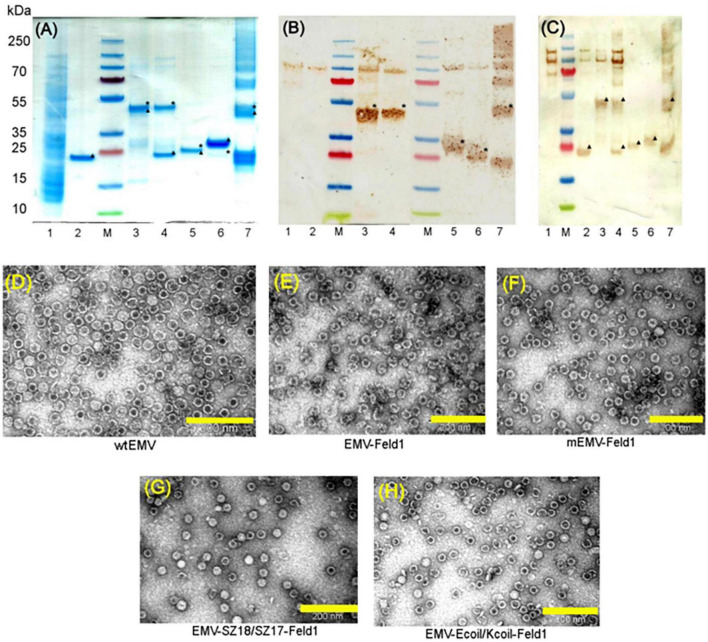
Analysis of EMV-derived VLPs containing Feld1 antigen. **(A)** Coomassie – stained 10 well 1.0 mm Bolt 4–12% Bis-Tris Plus gel (Thermo Fisher Scientific, USA) of EMV-Feld1 vaccine variants at a concentration of 0.7 mg/ml; **(B)** the same samples as in panel **(A)** analyzed by Western blot using anti-Feld1 polyclonal antibodies; and **(C)** the same samples as in panel **(A)** analyzed by Western blot using anti-EMV polyclonal antibodies. Electron micrographs of negatively stained VLPs: **(D)** EMV wt; **(E)** direct fusion EMV-Feld1; **(F)** mosaic EMV-Feld1; **(G)** EMV-SZ18/SZ17-Feld1; and **(H)** EMV-Kcoil/Ecoil-Feld1. Scale bars, 200 nm. M, PageRuler Plus Prestained Ladder, 10–250 kDa (Thermo Fisher Scientific, USA); 1, total cell lysate of *E. coli* C2566 strain (negative control); 2, wtEMV (control); 3, EMV-Feld1; 4, mEMV-Feld1; 5, EMV-SZ18/SZ17-Feld1; 6, EMV-Ecoil/Kcoil-Feld1; 7, wtEMV coupling with 2xFeld1-C6H-CG. Asterisk denotes the relative position of Feld1 protein; triangle denotes the relative position of EMV CP protein.

Next, we constructed direct fusion of EMV-CG4S and Feld1 antigen (Supplementary Figure 1) and expressed the fusion protein in *E. coli* cells. In addition, we constructed mosaic VLPs by incorporating unmodified wtEMV *CP* and the newly constructed EMV-CG4S-Feld1 *CP* gene into the pET-Duet expression vector (Supplementary Figure 2). After expression in *E. coli* cells, both EMV-CG4S-Feld1 CPs and mEMV-CG4S-Feld1 CPs were analyzed in SDS-PAGE, confirming an EMV-CG4S-Feld1 protein size of ∼50 kDa (Figure 2A, lane 3), but for the mosaic system, two bands were identified – the same of 50 kDa for EMV-CG4S-Feld1 CP and wtEMV CP with a size of ∼19.6 kDa (Figure 2A, lane 4), which was exactly the same as the separately expressed wtEMV used for control analysis (Figure 2A, lane 2). Electron microscopy analysis resulted in the identification of *Tymovirus*-like particle formation with a size of approximately 30 nm for both constructed VLP platforms. Unlike wtEMV VLPs (Figure 2D), Feld1-containing particles seemed more heterogeneous, and fragments of partially assembled VLPs were identified for either direct (Figure 2E) or mosaic VLPs (Figure 2F). In the case of direct fusion, each CP subunit is fused to the Feld1 antigen, resulting in 100% incorporation of the Feld1 antigen. The results of densitometric analysis of SDS-PAGE images suggested an approximate 46% Feld1 antigen incorporation in mosaic VLPs. The output of both VLPs after purification was approximately 7–8 mg/L.

To overcome the limiting factors that may arise from genetic fusion, such as incomplete assembly and stability of VLPs, several approaches using chemical and physical methods are available (Balke and Zeltins, 2019). We have previously described a successful strategy of using SpyTag/SpyCatcher for VLP-Feld1 chemical conjugate formation directly in bacterial cells (Ogrina et al., 2022). Here, we tested two binding partners: coiled coil-forming oligopeptide K and E interaction (Kcoil/Ecoil; Litowski and Hodges, 2002) and orthogonal synthetic coiled-coil anti-parallel “zipper” pair SYNZIP17/18 (SZ17/18; Reinke et al., 2010; Thompson et al., 2012), which should form stable physical complexes between protein partners when Kcoil/Ecoil or SYNZYP 17/18 sequences are genetically introduced in target proteins.

First, we constructed two EMV CP genetic fusions encoding DNAs of negatively charged peptides (Ecoil or SZ18, Supplementary Figures 3, 6) were subcloned into the pET42-EMV-CG4S CP plasmid, resulting in two new expression vectors coding for EMV-CG4S-Ecoil and EMV-CG4S-SZ18, which were then expressed separately in the *E. coli* cell strain C2566 to confirm solubility, expression level, and intact VLP formation (Supplementary Figures 8, 10). Further cloning involved the subcloning of the *Feld1* coding sequence into plasmids containing either the positively charged SZ17 or Kcoil sequence (Supplementary Figures 4, 5, 7). Both binding partners were then coexpressed, purified, and used for further characterization by SDS-PAGE, WB, and NAG analysis. To confirm that separately expressed and purified VLPs and Feld1 are capable of interacting, we also expressed each binding partner in the C2566 *E. coli* strain separately, followed by purification and binding. After binding, we observed complex formation after agarose gel analyses (Supplementary Figures 9, 11). The output of VLPs was approximately 77 mg/L for SZ18/17 and 46 mg/L for Ecoil/Kcoil coexpression VLPs.

Molecular mass calculations suggested that the SZ18/17 protein pair has an approximately similar molecular weight at ∼25 kDa, which can be seen as overlapping bands in SDS-PAGE (Figure 2A, lane 5), whereas the Ecoil/Kcoil protein pair appears as two distinct bands, where one could be identified as an ∼23 kDa band corresponding to the EMV-Ecoil part and a weak signal band at ∼ 21.7 kDa for the Feld1-Kcoil part (Figure 2A, lane 6). To confirm that both covalently linked pairs are present in our samples, we performed WB analysis using antibodies against both Fel d 1 and carrier EMV VLPs for all vaccine candidates, where wtEMV was used as a negative control for the Feld1 signal (Figures 2B, C, lane 2). In the case of Fel d 1, we used polyclonal Fel d 1 Abs obtained from our previous experiments with BALB/c mice (Zeltins et al., 2017). WB analysis indicated that both Feld1 and EMV were copurified in the same sucrose-gradient fractions (Figures 2B, C), seen as colored bands at the expected protein sizes when analyzed with corresponding antibodies (anti-Feld1 or anti-EMV). Furthermore, probably due to chosen purification conditions that failed to remove small amounts of host proteins, all of the samples gave additional ∼100 kDa bands (Figures 2B, C, lanes 1–6), which were considered the background, as some in-house-produced polyclonal Abs reacted to those proteins as well during previous immunization experiments.

For EMV detection in WB, we used polyclonal Abs from experiments with Balb/C mice immunized with wtEMV-derived VLPs (unpublished) and observed that EMV was present in all samples (Figure 2C). Densitometric analysis of SDS-PAGE gels suggested 56% Fel d 1 incorporation for EMV-SZ18/SZ17-Feld1 and only 5.2% for EMV-Kcoil/Ecoil-Feld1. Additional TEM analysis demonstrated icosahedral particle formation for both vaccine constructs (Figures 2G, H).

### 3.2. Chemical coupling of EMV CP with Feld1

**FIGURE 3:**
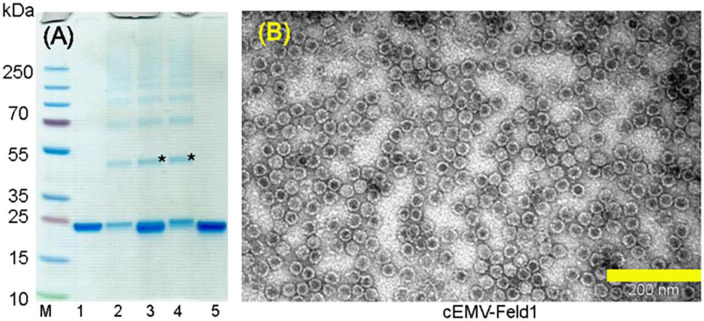
Chemically coupled EMV-2xFeld1 analysis. **(A)** Coomassie – stained 10 well 1.0 mm Bolt 4–12% Bis-Tris Plus gel (Thermo Fisher Scientific, USA). **(B)** Electron micrographs of negatively stained cEMV-Feld1. M, PageRuler Plus Prestained Ladder, 10–250 kDa (Thermo Fisher Scientific, USA); 1, wtEMV; 2, EMV after 3xSMPH derivatization and unreacted SMPH removal; 3, EMV coupling with Feld1-C6H-CG; 4, EMV + 2xFeld1-C6H-CG after Feld1-C6H-CG removal; 5, Feld1-C6H-CG treated with 10x TCEP. *Coupling product of EMV-rFeld1. Scale bar, 200 nm.

Two components (EMV CP VLP and antigen) for chemically coupled EMV-Feld1 (cEMV-Feld1) variant construction were expressed and purified separately. First, we purified unmodified wtEMV VLPs as described in the Materials and Methods and measured the concentration using a Qubit Protein Assay Kit (Thermo Fisher Scientific, USA). The purified sample was analyzed by SDS-PAGE (Figure 2A, lane 2 and Figure 3A, lane 1), and the correct protein size of ∼25 kDa was confirmed. TEM was used to visualize VLP formation (Figure 2D). The wtEMV sample was then mixed with SMPH for derivatization and incubated for 1 h, after which unreacted SMPH was removed.

Recombinant Fel d 1 protein with an additional His-tag was purified as previously described (Zeltins et al., 2017; Ogrina et al., 2022) followed by treatment with the reducing agent 10xTCEP to gain access to the cysteine at the COOH terminus of rFeld1. Finally, 2 molar excess of rFeld1-TCEP complex was added to derivatized EMV at 23°C for 2 h, and uncoupled rFeld1 was then removed by ultrafiltration in Amicon Ultra-0.5, 50 K (Merck-Millipore, USA) filtration units. The coupling reaction was monitored in an SDS-PAGE gel (Figure 3A), and the final product was observed by TEM (Figure 3B), representing well-assembled VLPs and preserving the *T* = 3 icosahedral structure. As the dimer of the EMV CP overlaps with the cEMV-Feld1 signal in the SDS-PAGE gel (Figure 3A, lane 4), we could not perform densitometric analysis of Feld1 incorporation, however, we were able to demonstrate the presence of the cEMV-Feld1 conjugate in WB analysis, which suggests that a successful reaction has taken place (Figures 2B, C, lane 7).

### 3.3. EMV-Feld1 VLP encapsidated nucleic acids and overall charge

*Tymoviruses* lack strong RNA-protein interactions due to missing specific regions rich in basic residues in CP amino acid composition (Dupin et al., 1985). As described above, during infection, *Tymoviruses* may produce two types of virions—one that contains a single viral genome (approximately 35% of their mass) as well as free “empty” protein shells, while some of those empty VLPs may pack small RNA molecules from their hosts (Gibbs and Mackenzie, 1998). Another interesting aspect of empty particle formation was addressed in a recent paper suggesting that freezing conditions may influence viral RNA migration through pores in *Tymovirus* capsids (Powell and Dreher, 2022). These observations stimulated us to evaluate encapsulated nucleic acids in all constructed EMV-Feld1 VLPs. This is a very important aspect for vaccine generation, as incorporated nucleic acids serve as Toll-like receptor (TLR) ligands and improve the overall immune response (Mohsen et al., 2020).

**FIGURE 4:**
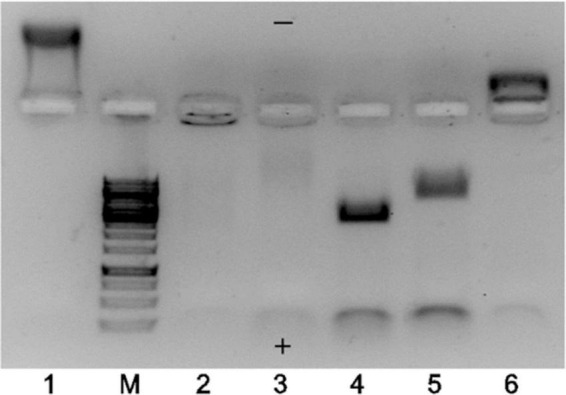
Agarose gel analysis of EMV–Feld1 vaccine variants at a concentration of 0.7 mg/ml. 1, wtEMV (wild-type; control); M, GeneRuler 1 kb DNA ladder (Thermo Fisher Scientific, USA); 2, EMV-Feld1 (direct fusion VLPs); 3, mEMV-Feld1 (mosaic VLPs); 4, EMV-SZ18/SZ17-Feld1; 5, EMV-Ecoil/Kcoil-Feld1; 6, cEMV-Feld1 (chemically coupled). A 0.8% native agarose gel stained with ethidium bromide was used for analysis. The symbols “+” and “−” denotes the electrode polarity.

The TEM results strongly indicated that the majority of EMV VLPs produced in *E. coli* were indeed “empty” despite using different construction strategies, however, the total proportion of “full” particles increased in the case of binding pairs SZ18/SZ17 and Ecoil/Kcoil as well as for chemically coupled EMV-Feld1 VLPs. To explore this further, we performed native agarose gel (NAG) analysis of constructed vaccines with ethidium bromide staining to evaluate whether the results of TEM analysis correlated with their nucleic acid content. We concluded that the signal intensity for physical binding pairs and chemically coupled VLPs was much stronger than that for direct fusion and mosaic VLPs, as expected (Figure 4, lanes 4, 5, and 6).

Another finding after agarose gel analysis was a different movement of VLPs in the electric field. We observed wtEMV migration toward the negative electrode (Figure 4, lane 1) in the selected buffer system, which can be explained by the calculated isoelectric point of EMV CP (*IEP* = 8.52). The incorporation of Fel d 1 in all VLPs resulted in a reduction in the VLP positive charge due to negatively charged Fel d 1 molecules. Interestingly, direct and mosaic fusion VLPs contained significantly less host RNA than all other vaccine variants (Figure 4, lanes 2 and 3). We also observed that EMV-SZ18/SZ17-Feld1 and EMV-Ecoil/Kcoil-Feld1 VLPs were strongly negatively charged (Figure 4, lanes 4 and 5) and contained large amounts of host nucleic acids.

Our experiments show that a higher nucleic acid content results in VLPs with a higher proportion of “filled” particles. Furthermore, the chosen antigen and nucleic acid content can affect the overall VLP charge. It is known that the surface charge of nanoparticles plays a significant role in the modulation of the immune response, particularly in the activation of dendritic cells (DCs). Moreover, positively charged nanoparticles considerably increase interleukin expression and secretion by DCs compared to negatively charged particles (Fontana et al., 2019).

### 3.4. Immunological characterization of the EMV-Feld1 vaccine candidates

To gain insight into the immune stimulation properties of the constructed vaccine variants, we performed immunization experiments using murine models. First, before animal immunizations, we evaluated the antigenicity of introduced model antigens on the VLP surface by ELISA using commercially available monoclonal antibodies against Fel d 1 (MA-6F9; MA-3E4; Indoor Biotechnologies, UK). The constructed and purified vaccines were injected into mice, and the formation of total IgG was monitored during repeated vaccination. Additionally, we tested subclass-specific IgG1 and IgG2 Abs and the avidity of Abs obtained from Day 42 to elucidate the proportion of strongly binding antibodies, which correlates with vaccine efficacy, as confirmed in previous studies (Dobano et al., 2019; Rothen et al., 2022). Additionally, the specificity of the generated polyclonal Abs was tested against both recombinant and native Fel d 1 antigen and compared with commercially available mAbs. Immune responses and Ab avidity results are considered important criteria for choosing the most effective VLP variant for further VLP platform development.

#### 3.4.1. Monoclonal antibody ELISA

**FIGURE 5:**
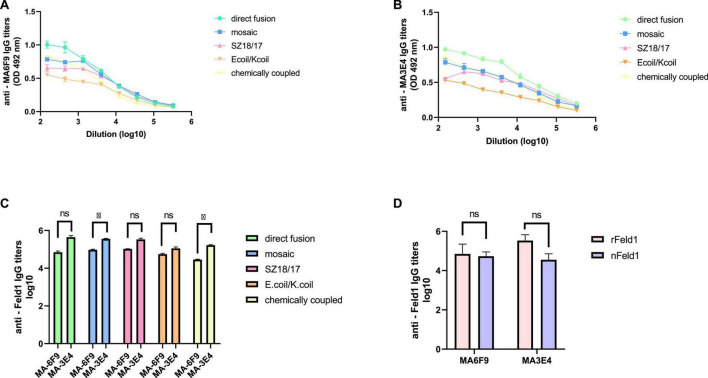
Monoclonal Feld1 antibody titers with displayed Feld1 on the surface of vaccine variants. Plates were coated with 10 μg/ml of each VLP. **(A)** Total IgG titers against Feld1 monoclonal antibody MA-6F9 (Indoor Biotechnologies, UK); **(B)** total IgG titers against Feld1 monoclonal antibody MA-3E4 (Indoor Biotechnologies, UK); **(C)** comparison of MA-6F9 and MA-3E4 titers against each vaccine construct; and **(D)** comparison of monoclonal antibody MA-6F9 and MA-3E4 titers against recombinant Feld1 (rFeld1) or native Feld1 (nFeld1). **(C,D)** Statistical analysis using Student’s *t*-test in GraphPad Prism 8.0. Vaccine groups *n* = 5. Graphs are showing the mean values of single titers. A value of *p* > 0.05 was considered statistically significant (*p < 0.01).

To characterize the immunogenicity of the constructed EMV-Feld1 vaccine variants, we used two commercially available Fel d 1 mAbs in ELISA experiments as previously described (Ogrina et al., 2022). First, we compared nFel d 1 and rFel d 1 to determine whether rFel d 1 incorporated into VLP structures can be recognized as native. Interestingly, according to the obtained results, rFel d 1 is recognized even better than nFel d 1 by either MA-6F9 or MA-3E4 (Figure 5D), although both mAbs are produced against native Feld 1 allergen. The same finding was detected in our previously performed experiments (Ogrina et al., 2022), but otherwise, no significant differences between mAb titers were identified.

Although both mAbs (MA-3E4; MA-6F9; Indoor Biotechnology, USA) were produced specifically against chain-1 of cat *F. domesticus* allergen Fel d 1 (Schmitz et al., 2009), our recent data with potato virus Y (PVY)-Fel d 1-based vaccines revealed that the binding ability of MA-3E4 mAb was more efficient than MA-6F9 mAb for all produced variants, possibly due to the higher specificity and better access to the corresponding part of the exposed Fel d 1 epitope from chain 1 (Ogrina et al., 2022). The data presented in this experiment also indicated that titers for MA-3E4 mAbs were at least twice as high as those for MA-6F9 for all EMV-Feld1 vaccine variants tested, reaching the maximum for EMV-Feld1 direct fusion (corresponding reciprocal titers for MA-6F9 1: 71,000; for MA-3E4 1: 447,000) (Figures 5A–C) and minimum for EMV-Ecoil/Kcoil-Feld1 (corresponding reciprocal titers for MA-6F9 1: 29,000; for MA-3E4 1: 168,000). Furthermore, both mAbs were also highly specific to mEMV-Feld1 and EMV-SZ18/SZ17-Feld1 (reciprocal titers varying between ∼ 1: 350,000 for MA-6F9 and ∼ 1: 100,000 for MA-3E4), confirming the presentation of the Fel d 1 epitope on the EMV VLP surface. Overall, these mAb results indicate the most promising VLP vaccine platform with the correct folding of the Fel d 1 epitope after introduction into EMV VLPs.

#### 3.4.2. Total IgG levels

Only some *Tymovirus*-based vaccine studies have emphasized the high potential of the well-described PhMV VLP platforms as effective vaccine candidates either by testing their immunogenicity in sera (Hema et al., 2007; Sahithi et al., 2019) or by performing immunization experiments in guinea pigs and dogs (Chandran et al., 2009) and mice (Shahana et al., 2015; Hu and Steinmetz, 2021). To evaluate the immunological potential of the EMV-based vaccines, total IgG levels against both principal components—antigen Fel d 1 and EMV VLPs of produced chimeric VLP vaccines—were measured by ELISA analysis in murine sera collected on Days 14, 28, and 42.

**FIGURE 6:**
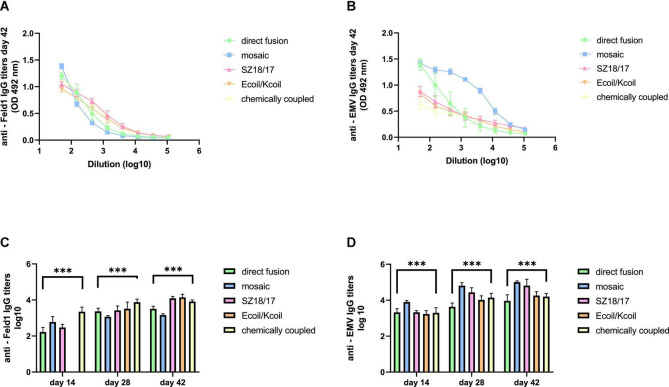
IgG titer analysis after vaccination with EMV-Feld1 vaccine variants. **(A)** Feld 1 specific IgG titers on Day 42 for the groups immunized with EMV-Feld1 variants measured at OD 492 nm; **(B)** EMV CP-specific IgG titers on Day 42 for the groups immunized with EMV-Feld1 variants measured at OD 492 nm; **(C)** Log_10_ values (mean ± SEM, *n* = 5) of Feld1-specific IgG titers for the groups vaccinated with EMV-Feld1 VLPs on Days 14, 28, and 42; **(D)** Log_10_ values (mean ± SEM, *n* = 5) of EMV-specific IgG titers for the groups vaccinated with EMV-Feld1 VLPs on Days 14, 28, and 42. **(C,D)** Statistical analysis using one-way ANOVA in GraphPad Prism 8.0. Vaccine groups *n* = 5. Graphs are showing the mean values of single titers. A value of *p* > 0.05 was considered statistically significant (***p < 0.0001).

First, we noticed that the majority of produced EMV-Feld 1 vaccines induced Fel d 1-specific IgG production on Day 14, reaching the highest titers for the cEMV-Feld1 variant (reciprocal titers 1:2,800; Figure 6C; Supplementary Figure 12), whereas Feld 1-specific antibodies for the Ecoil/Kcoil vaccine were not yet detected (Figure 6C), probably due to the low Feld1 incorporation level discussed previously. Following the booster dosage on Day 14 Fel d 1, the specific IgG titers measured on Day 28 increased by at least 2 times for mEMV-Feld1 (reciprocal titers 1: 2,500; Figure 6C), while a minimum of fourfold excess was detected for the other four vaccine candidates, demonstrating that effective immunostimulation through B-cell activation was accomplished (Bachmann and Jennings, 2010). Furthermore, an additional booster dose dramatically prompted the Feld1-specific antibody response in mice vaccinated with the Ecoil/Kcoil variant (corresponding reciprocal titers 1: 3,900; Figure 6C) as well as for EMV-Feld1 (corresponding reciprocal titers 1: 2,400; Figure 6C), although cEMV-Feld1 once again demonstrated the highest Fel d 1-specific IgG response on Day 42 among all tested. Data presented for the final IgG titers on Day 42 after the second booster showed differences in some results—the most effective Fel d 1-specific antibody elicitors were SZ18/17 and Ecoil/Kcoil vaccine variants (corresponding reciprocal titers approximately 1: 12,000 for both; Figures 6A, C) despite the relatively long delay in anti-Fel d 1 Ab production for the Ecoil/Kcoil variant. Unfortunately, EMV-Feld1 and mEMV-Feld1 did not elicit high IgG levels, as their titers varied between 1:3,400 for direct fusion and 1:1,500 for the mosaic variant even after two booster injections (Figures 6A, C).

Interestingly, all measurements for EMV-specific IgG titers demonstrated a much higher IgG response against VLP carriers than against Feld 1-specific Abs (Figure 6D; Supplementary Figure 12). The same tendency was observed throughout the experiment, meaning that VLPs themselves have a crucial effect on triggering strong immune responses. For instance, of all vaccine constructs tested, titers against EMV were the highest for mEMV-Fel d 1 at every time point measured (Days 14, 28, and 42), reaching 1:68,000 after the first booster (Day 28) and increasing even more after the second on Day 42 (corresponding reciprocal titer 1:102,000; Figures 6B, D). Anti-EMV titers dramatically exceeded anti-Feld1 titers in three out of five groups (EMV-Feld1, mEMV-Feld1, SZ18/17) in vaccinated mouse sera obtained from Day 42 producing 1:12,000 for EMV-Fel d 1 and 1:66,000 for SZ18/17. Interestingly, similar observations were shown by Hu and Steinmetz in 2021 using a PhMV-based cancer vaccine, where anti-PhMV titers were higher than those against the chemically coupled HER (human epidermal growth factor receptor)-2-derived CH401 epitope (Hu and Steinmetz, 2021). This leads to the speculation that some *Tymovirus*-like particles strongly resemble a native virus through particulate virus-like structures and are able to stimulate the immune system more efficiently than surface-exposed Fel d 1 antigen (Mohsen et al., 2017). Here, additional studies are needed.

Our recent study regarding the MERS-coronavirus (CoV) VLP vaccine in murine immunological experiments showed that the production of specific antibodies with a high avidity index successfully provided protection against infectious diseases such as MERS-CoV (Mohsen et al., 2021). As the higher avidity index may correlate with efficient Fel d 1 neutralization, we evaluated the antibody avidity for all five EMV-Feld1 vaccine variants.

**FIGURE 7:**
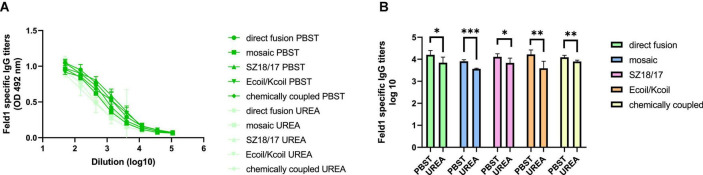
Avidity antibody detection after vaccination with EMV-Feld1 vaccine variants. **(A)** Feld1 specific IgG titers on Day 42 for the groups vaccinated with EMV-Feld1 variants measured with OD_492_ nm. After serum incubation one plate was treated with PBS with 0.05% Tween-80 and the other plate with 7 M UREA in PBS with 0.05% Tween-80; **(B)** Log_10_ values (mean ± SEM, *n* = 5) of Feld1 specific IgG titers (shown in panel **A**) for the groups vaccinated with EMV-Feld1 vaccine variants. Statistical analysis using Student’s *t*-test in GraphPad Prism 9.0. Vaccine groups *n* = 5. Graphs are showing the mean values of single titers. A value of *p* > 0.05 was considered statistically significant (*p < 0.01; **p < 0.001; ***p < 0.0001).

We compared two identical ELISA plates in the total IgG detection experiment, where one plate was treated with an additional 7 M UREA washing step and the other plate was treated as usual (Ogrina et al., 2022) to determine Ab binding avidity. The results showed that four out of five vaccine groups produced Abs with very high avidity index (AI) values (Figures 7A, B; Supplementary Figure 13), resulting in over 50% binding specificity represented as a proportion of Abs still bound to antigen after a 7 M UREA washing step. Three of the vaccines with the highest avidity were SZ18/17, cEMV-Feld1, and Ecoil/Kcoil, all reaching 64–68% specificity, whereas mEMV-Feld1 resulted in less specific Ab production (47%), suggesting that the most promising vaccine for future experiments should be chosen from candidates demonstrating the highest AI values.

#### 3.4.3. Subclass specific IgG production

Different approaches should be considered when shaping the most beneficial immune response, as the strategy used for anti-pathogen vaccines can be not as successful against allergies and vice versa (Zepeda-Cervantes et al., 2020). Modern solutions in allergy treatment now include not only widely described allergen-specific IgE-targeted treatments but also novel allergen-specific IgGs, which are possible key effectors for so-called next-generation immunotherapy when allergens are displayed on the VLP surface (Schmitz et al., 2009; Bachmann et al., 2020). A recent paper underlines that specific IgG subclasses play a minor role in allergy-specific immunotherapy (Zinkhan et al., 2022).

It has been emphasized that strong activators of antigen presenting cells (APC) such as VLPs are recognized by so-called pathogen recognition receptors (PRRs) expressed on the surface of antigen-presenting cells (APCs). Specific types of PRRs are TLRs, which are able to recognize various viral or bacterial pathogen-associated molecules. After TLR stimulation, these VLPs are taken up and processed by the most potent APCs, dendritic cells (DCs), leading to their maturation and further proinflammatory responses (Zepeda-Cervantes et al., 2020). Captured antigens are then presented on the surface of mature DCs for T-cell recognition through major histocompatibility complex (MHC) classes I or II and through cross-presentation (Mohsen et al., 2017; Zepeda-Cervantes et al., 2020), which then determine the IgG isotype pattern. In the case of VLP-based vaccines, two mural antibody subclasses (IgG1 and IgG2a) are linked to Th2 and Th1 signaling pathways, each representing different effector functions. Hence, the immune response driven by Th1 is more defensive against viral infections (Nimmerjahn and Ravetch, 2008; Gomes et al., 2019) and may help to control IgE-dependent allergic reactions by regulating allergen-specific IgG production (Bachmann and Kündig, 2017).

**FIGURE 8:**
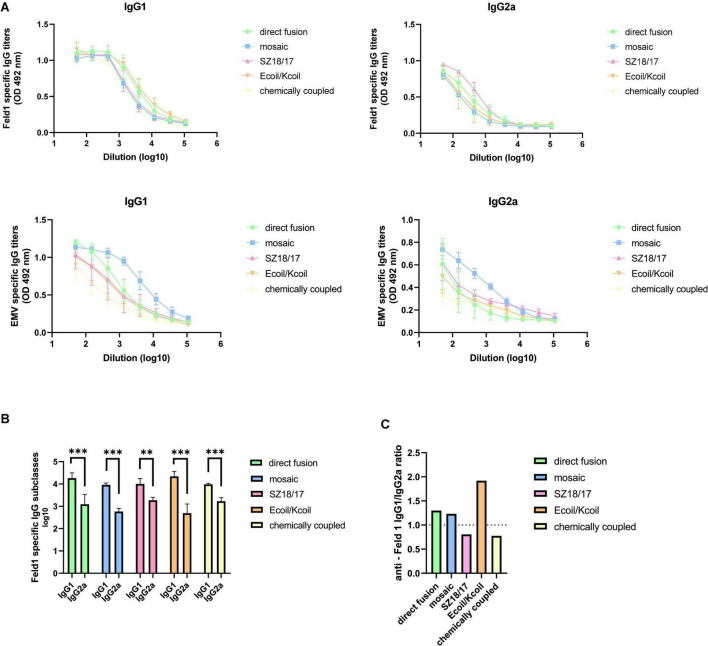
Subclass specific antibody detection. **(A)** Anti-Feld1 and anti-EMV CP specific IgG1 and IgG2a titers measured in Day 42 mice sera using OD 492 nm. ELISA plates were coated with EMV-Feld1 variants for IgG subclass detection in mice sera vaccinated with EMV-Feld1 vaccine variants. **(B)** Log_10_ values of Feld1 specific IgG1 and IgG2a titers measured in Day 42 mice sera; **(C)** anti-Feld1 IgG1/IgG2a ratio measured from mean titers used in experiment shown in panel **(B)**. **(A,B)** Statistical analysis using Student’s *t*-test in GraphPad Prism 8.0. Vaccine groups *n* = 5. Graphs are showing the mean values of single titers. A value of *p* > 0.05 was considered statistically significant (**p < 0.001; ***p < 0.0001).

To characterize the nature of the generated immune response, we measured IgG1 and IgG2a titers in mouse sera collected on Day 42 against both the Fel d 1 antigen and EMV VLPs. Our obtained data clearly demonstrated IgG1 subclass dominance for all variants tested, producing the highest anti-Feld1 IgG1 titers for Ecoil/Kcoil and EMV-Feld1 (corresponding reciprocal titers 1: 25,000 for Ecoil/Kcoil; 1: 20,500 for EMV-Feld1; Figures 8A, B; Supplementary Figure 14), whereas the highest IgG2a antibody response was approximately similar for three groups of EMV-Feld1, SZ18/17, and cEMV-Feld1, producing titers of 1: 1,700–2,000, respectively (Figures 8A, B). Additionally, the subclass-specific Ab response against carrier EMV was dominated by IgG1 producing the highest titers for mEMV-Feld1 (reciprocal IgG1 titers 1: 56,000; IgG2a titers 1: 5,800; Figures 8A, B).

Furthermore, we also determined that the lowest Feld1 IgG1/IgG2a ratio values were obtained for SZ18/17 and cEMV-Feld1 (ratio values <0.8) (Figure 8C), meaning that these vaccine candidates induce more balanced Th1/Th2 phenotypes when compared with Ecoil/Kcoil (ratio value 1.92) and therefore should be considered the most promising candidates for desired protective immune response generation in anti-allergy treatment procedures.

Virus-like particles induce an immune response dominated by the Th2 pathway producing IgG1 Abs unless TLR ligands such as nucleic acids are present that may achieve isotype switching toward the IgG2a Ab response (Jegerlehner et al., 2007). As IgG1 predominance was also addressed in other studies with VLP-based vaccines (Cai et al., 2019; Hu and Steinmetz, 2021; Ogrina et al., 2022), we assumed that TLR stimulation was not sufficiently effective due to the reduced amount of encapsidated nucleic acids, as observed in NAG analysis. This observation possibly indicates the low activation of TLR7/8 dependent responses (Gomes et al., 2019; Krueger et al., 2019).

#### 3.4.4. Native Fel d 1 recognition

Allergy cases caused by the domestic cat *F. domesticus* arise each year, affecting a large part of the population, as common symptoms such as allergic respiratory diseases negatively affect quality of life (Bonnet et al., 2018). Recent studies in the VLP field have highlighted the optimal strategy for allergy treatment focusing on neutralizing IgG Ab production when challenged with native Fel d 1 allergen (Schmitz et al., 2009; Thoms et al., 2019) or peanut allergy allergens Arah1 and Arah2 (Storni et al., 2019). Therefore, we evaluated the recognition of native Fel d 1 by antibodies obtained after mouse vaccination with all constructed vaccine variants using native Fel d 1 (nFel d 1; Indoor Biotechnology, USA).

**FIGURE 9:**
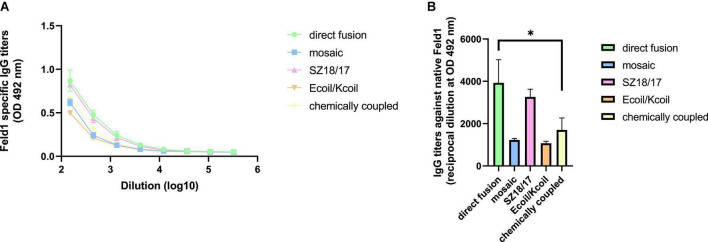
Native Feld1-specific antibody detection in EMV-Feld1 vaccinated mice sera. **(A,B)** Feld1 specific IgG titers (mean ± SEM) against native Feld1 for the groups vaccinated with EMV-Feld1 vaccine variants. Sera obtained from Day 42 and measured at OD 492 nm. **(B)** Statistical analysis using one-way ANOVA in GraphPad Prism 8.0. Vaccine groups *n* = 5. Graphs are presenting mean of pooled mice titers. A value of *p* > 0.05 was considered statistically significant (*p < 0.01).

The obtained results revealed that titer values for EMV-Feld1 reached almost 1:4,000 and represented the highest IgG titers against nFel d 1 (Figures 9A, B) but were closely followed by SZ18/17 (corresponding reciprocal titers 1:3,300; Figures 9A, B), whereas the lowest anti-Feld1 IgG titers were detected for the Ecoil/Kcoil and mEMV-Feld1 vaccines, which varied by approximately 1:1,000. These results correlate with avidity measurements, where the SZ18/17 variant produced the most specific Abs against Feld1 compared to other vaccine candidates. Interestingly, we have noted that rFeld1 used for cloning experiments and covering ELISA plates is somehow recognized more efficiently than the native form, which was also observed for mAb ELISA experiments. Furthermore, titers against nFel d 1 were significantly lower for EMV-derived Feld1 vaccine constructs than for filamentous Feld1 antigen-containing PVY CP VLPs (Ogrina et al., 2022), meaning that despite the large size of the 200–400 nm filaments of PVY CP VLPs, they could represent the Fel d 1 antigen in a much more recognizable manner, observed as high levels of nFeld1 binding IgGs.

## 4. Conclusion

The generation of more effective vaccine candidates can improve vaccine manufacturing by avoiding complicated, multistep processes in vaccine construction and purification. As the native spatial structures of recombinant antigens are highly important for eliciting antibodies with neutralizing activity and long-lasting immunity, recombinant VLP vaccines must contain strongly organized and multiple copies of exposed antigen (Balke and Zeltins, 2019). Here, we demonstrated the construction and tests of several simple bacterial expression systems for vaccine generation based on new *Tymovirus*-like particles and different principles of Fel d 1 antigen incorporation, including genetic techniques by introducing the antigen directly into EMV CP or as a part of a mosaic system with wtEMV CP. Additionally, we showed two physical binding approaches resulting in VLP-antigen complexes using synthetic zipper pairs 18/17 (SZ18/17) and coiled-coil-forming peptides E and K (Ecoil/Kcoil). Both systems were tested *in vitro* after purification of the corresponding proteins and in coexpression directly in *E. coli* cells. These experiments resulted in the formation of stable complexes regardless of the method of epitope incorporation. Here, we have demonstrated that these protein–protein interaction partners allowed the binding of coexpressed antigen to the VLP surface directly in *E. coli* cells during cultivation due to their ability to form highly specific and high affinity interactions with a low dissociation constant (*K*_d_ < 1 × 10^–8^; Reinke et al., 2010; Thompson et al., 2012). The presented systems can be used for the introduction of antigens, resulting in new antigen presentation platforms, as they reduce the influence of expressed antigens on the spatial structure and self-assembly of VLPs.

All four constructed systems were successfully expressed in *E. coli* and purified in the form of antigen-containing VLPs. The presence of EMV CP VLPs and Feld1 antigen for all four vaccine variants was confirmed by SDS-PAGE, WB, and ELISA using mAbs. As the *E. coli* expression systems allow rapid expression and subsequent large-scale, cost-effective manufacturing of recombinant proteins, the constructed vaccines can be a prototype for Feld1 therapeutic vaccines in the future. Chemical coupling of Feld1 to wtEMV CP VLPs was also achieved and therefore included in immunological experiments, resulting in a total of five different EMV-Feld1 vaccine variants tested in murine models.

To our knowledge, this is the first report of successful tymoviral VLP formation after the introduction of long antigen sequences into the C-terminal end of CP. Our experiments suggest that EMV CP VLPs are fully suitable for CP modifications and ensure the necessary VLP morphology. Furthermore, obtained immune responses, antibody avidity, and subclass switching toward the Th1 pathway were the main criteria for choosing the most effective EMV-Feld1 VLP platform. Therefore, we conclude that cEMV-Feld1 and SZ18/17 should be considered the most efficient platforms for antigen presentation, as they possess most of the features needed. All five constructed vaccines elicited high titers of Feld1-specific Abs. Moreover, the obtained antibodies recognized the native Fel d 1 allergen; therefore, our results suggest good potential for SZ18/17 and cEMV-Feld1 vaccine variants as the most promising VLP platforms among all tested.

However, the vaccine’s ability to produce comparatively higher titers against carrier EMV is a matter of concern. In the context of emerging vaccination strategies, an ideal vaccine candidate should be universal, meaning that one antigen presentation platform should be adapted for multiple vaccine development. In the case of EMV VLPs, their usage can be limited due to their high immunogenicity against the VLP carrier. Another aspect that should be taken into consideration is that the constructed EMV-Feld1 VLPs produce low levels of protective IgG2a subclass antibodies, which correlates with the reduced nucleic acid signal intensity detected in NAG and the “empty” particle presence observed in TEM.

The induction of optimal B-cell responses depends on immunostimulating characteristics such as repetitive epitope structures spaced 5–10 nm from each other on the VLP exterior and surface charge that can attract the protein corona consisting of absorbed serum proteins and natural antibodies (Blanco et al., 2015). Another factor is the size and morphology of vaccine carriers, suggesting that antigens larger than 200 nm struggle to freely enter the lymphatic system when compared to icosahedral particles with a size of ∼30 nm (Mohsen et al., 2017; Zinkhan et al., 2021). As we have previously described similar Fel d 1 epitope presenting strategies based on bacterial expression platforms using filamentous PVY VLPs (Ogrina et al., 2022), the results from this study gave us the opportunity to compare and evaluate icosahedral and filamentous VLPs and speculate which could serve as the most suitable candidate for vaccination purposes. Although both PVY-Feld1 and EMV-Feld1 vaccines elicited approximately similar total Ab levels against Fel d 1 (∼ 10^4^–10^5^), four EMV-Feld1 vaccine candidates stimulated a stronger Th1 immune response and IgG2a subclass production. Furthermore, the avidity of Abs produced was higher for EMV-Feld1 vaccine variants, meaning that ∼64–68% of Abs were very specific to surface-exposed Feld1 compared to 36% specificity for the best variant of PVY-Feld1 (Ogrina et al., 2022). On the other hand, better native Fel d 1 recognition was observed for Feld1 presenting PVY filaments; therefore, more comprehensive analysis between both strategies is needed.

Finally, the results of our designed and tested EMV-Feld1 vaccine platforms suggest that the chosen construction strategy can significantly influence the amplitude progress and specificity of the elicited immune responses. Therefore, several antigen incorporation strategies should be elaborated for the generation of each new VLP-based vaccine to find the best variant.

## Data availability statement

The original contributions presented in this study are included in the article/Supplementary material, further inquiries can be directed to the corresponding author.

## Ethics statement

This animal study was reviewed and approved by the Animal Protection Ethical Committee of the Latvian Food and Veterinary Service (permission no. 89).

## Author contributions

AO, MB, and AZ: conceptualization. AO, IB, IK, DS, and JJ: experimental work. AO, IB, and AZ: writing—original draft preparation. AO, IB, MB, and AZ: writing—review and editing. IB and AZ: supervision. AO and AZ: funding acquisition. All authors read and agreed to the published version of the manuscript.
