# Supplementary material for: Bacterial expression systems based on Tymovirus-like particles for the presentation of vaccine antigens
Source: Front Microbiol. 2023 Mar 23;14:1154990. doi: 10.3389/fmicb.2023.1154990 (PMC10076540; doi:10.3389/fmicb.2023.1154990)
Supplement: Supplementary file 1 [file Table_1.DOCX]

medtaiirspqpsinapgfhlpptdsqqssaielpfqfqattfgatetaaqislasanaitklaslyrhvrltqcaatitptaaaianpltvnivwvsdnstakpteilnvfggssytfggalnatkpltiplpmnsvncmlkdsvlytdcpkllaysaapsspsktptatiqihgklrlsspllqanggggsggggsggggseicpavkrdvdlfltgtpdeyveqvaqykalpvvlenarilkncvdakmteedkenalsvldkiytsplcggggsggggsggggsvkmaetcpifydvffavangnellldlsltkvnatepertamkkiqdcyvenglisrvldglvmttissskdcmgeavqntvedlklntlgr

Fel d 1: pI = 4.57; 18.9 kDa

EMV-CG4S-Feld1: pI = 5.14; MW = 39.6 kDa

**Suppl. Fig. 1.** Description of plasmid pET42-EMV-CG4S-Feld1. The expression vector ensures the synthesis of EMV-Feld1 fusion protein (direct fusion).

A – plasmid map with single-cut restriction enzyme sites;

B –amino acid sequence of EMV-CG4S-Feld1. EMV – CG4S AA are shown in black; Gly-Ser linkers – in green; Feld1 AA are shown in blue.

mEdtaiirspqpsinapgfhlpptdsqqssaielpfqfqattfgatetaaqislasanaitklaslyrhvrltqcaatitptaaaianpltvnivwvsdnstakpteilnvfggssytfggalnatkpltiplpmnsvncmlkdsvlytdcpkllaysaapsspsktptatiqihgklrlsspllqan

EMV – CP: pI – 8.52; Mw = 19.6 kDa.

mEdtaiirspqpsinapgfhlpptdsqqssaielpfqfqattfgatetaaqislasanaitklaslyrhvrltqcaatitptaaaianpltvnivwvsdnstakpteilNvfggssytfggalnatkpltiplpmnsvncmlkdsvlytdcpkllaysaapsspsktptatiqihgklrlsspllqanggggsggggsggggseicpavkrdvdLfltgtpdeyveqvaqykalpvvlenarilkncvdakmteedkenalsvldkiytsplcggggsggggsggggsvkmaetcpifydvffavangnellldlsltkvnatepertamkkiqdcyvenglisrvldglvmttissskdcmgeavqntvedlklntlgr**

Fel d 1: pI = 4.57; 18.9 kDa

EMV-CG4S-Feld1: pI = 5.14; MW = 39.6 kDa

**Suppl. Fig. 2.** Description of plasmid pET-Du-EMV-EMV-CG4S-Feld1. The expression vector ensures simultaneous synthesis of unmodified EMV CP and EMV-Feld1 fusion protein, resulting in mosaic VLPs.

A – plasmid map with single-cut restriction enzyme sites;

B –amino acid sequence of EMV-CP and EMV-CG4S-Feld1. EMV CP AA sequence are shown in black; Gly-Ser linkers - in green; Feld1 AA are shown in blue.

mEdtaiirspqpsinapgfhlpptdsqqssaielpfqfqattfgatetaaqislasanaitklaslyrhvrltqcaatitptaaaianpltvnivwvsdnstakpteilnvfggssytfggalnatkpltiplpmnsvncmlkdsvlytdcpkllaysaapsspsktptatiqihgklrlsspllqan

EMV – CP: pI – 8.52; Mw = 19.6 kDa.

mEdtaiirspqpsinapgfhlpptdsqqssaielpfqfqattfgatetaaqislasanaitklaslyrhvrltqcaatitptaaaianpltvnivwvsdnstakpteilNvfggssytfggalnatkpltiplpmnsvncmlkdsvlytdcpkllaysaapsspsktptatiqihgklrlsspllqanggggsggggsggggseicpavkrdvdLfltgtpdeyveqvaqykalpvvlenarilkncvdakmteedkenalsvldkiytsplcggggsggggsggggsvkmaetcpifydvffavangnellldlsltkvnatepertamkkiqdcyvenglisrvldglvmttissskdcmgeavqntvedlklntlgr**

Fel d 1: pI = 4.57; 18.9 kDa

EMV-CG4S-Feld1: pI = 5.14; MW = 39.6 kDa

**Suppl. Fig. 3.** Description of plasmid pET-Duet-EMV-CG4S-SZ18. The expression vector ensures the synthesis of EMV-CG4S-SZ18 protein.

A – plasmid map with single-cut restriction enzyme sites;

B –amino acid (AA) sequences of EMV-CP-CG4S-SZ18. EMV-CG4S AA are shown in black; Gly-Ser linkers - in green, SZ18 AA are shown in red.

medtaiirspqpsinapgfhlpptdsqqssaielpfqfqattfgatetaaqislasanaitklaslyrhvrltqcaatitptaaaianpltvnivwvsdnstakpteilnvfggssytfggalnatkpltiplpmnsvncmlkdsvlytdcpkllaysaapsspsktptatiqihgklrlsspllqanggggsggggsggggssiaatlendlarlenenarlekdianlerdlaklereeayf

EMV – CG4S: pI – 8.52; Mw = 20.7 kDa

SZ18: pI 4.44; Mw = 4.75 kDa

**Suppl. Fig. 4.** Description of plasmid pRSF-SZ17-Feld1. The expression vector ensures the synthesis of SZ17-Feld1 protein.

A – plasmid map with single-cut restriction enzyme sites;

B –amino acid (AA) sequences of SZ17-Feld1. SZ17 AA are shown in dark violet; Feld1 AA are shown in blue; G4S AA are shown in green.

MGSnekeelkskkaelrnrieqlkqkreqlkqkianlrkeieaykGSGGGGSeicpavkrdvdlfltgtpdeyveqvaqykalpvvlenarilkncvdakmteedkenalsvldkiytsplcggggsggggsggggsvkmaetcpifydvffavangnellldlsltkvnatepertamkkiqdcyvenglisrvldglvmttissskdcmgeavqntvedlklntlgr

SZ17: pI – 9.92; Mw – 5.18 kDa.

Fel d 1: pI = 4.57; 18.9 kDa.

MGSnekeelkskkaelrnrieqlkqkreqlkqkianlrkeieaykGSGGGGSeicpavkrdvdlfltgtpdeyveqvaqykalpvvlenarilkncvdakmteedkenalsvldkiytsplcggggsggggsggggsvkmaetcpifydvffavangnellldlsltkvnatepertamkkiqdcyvenglisrvldglvmttissskdcmgeavqntvedlklntlgrhhhhhhggcg

SZ17: pI – 9.92; Mw – 5.18 kDa.

Fel d 1- C6H: pI = 5.08; Mw – 20 kDa.

**Suppl. Fig. 5.** Description of plasmid pRSF-SZ17-Feld1-C6H. The expression vector ensures the synthesis of SZ17-Feld1-C6H protein.

A – plasmid map with single-cut restriction enzyme sites;

B –amino acid (AA) sequences of SZ17-Feld1 – C6H. SZ17 AA are shown in dark violet; Feld1-C6H AA are shown in blue; G4S AA are shown in green; 6xHis AA are shown in yellow.

**Suppl. Fig. 6.** Description of plasmid pET42-EMV-CG4S-Ecoil3x. The expression vector ensures the synthesis of EMV-CG4S-Ecoil3x protein.

A – plasmid map with single-cut restriction enzyme sites;

B –amino acid (AA) sequences of EMV-CG4S-Ecoil3x. EMV AA are shown in black; G4S AA are shown in green; Ecoil3x are shown in orange.

medtaiirspqpsinapgfhlpptdsqqssaielpfqfqattfgatetaaqislasanaitklaslyrhvrltqcaatitptaaaianpltvnivwvsdnstakpteilnvfggssytfggalnatkpltiplpmnsvncmlkdsvlytdcpkllaysaapsspsktptatiqihgklrlsspllqanggggsggggsggggs eiaalekeiaalekeiaalek

EMV-Cg4s: pI – 8.52; Mw – 20.7 kDa.

3xEcoil: pI – 4.48; Mw – 2.28 kDa.

**Suppl. Fig. 7.** Description of plasmid pACYC-Kcoil3x-Feld1. The expression vector ensures the synthesis of Kcoil3x-Feld1 protein.

A – plasmid map with single-cut restriction enzyme sites;

B –amino acid (AA) sequences of Kcoil3x. 3xKcoil AA are shown in pink; Feld1 are shown in blue; G4S AA are shown in green.

MGkiaalkekiaalkekiaalkegsmeicpavkrdvdlfltgtpdeyveqvaqykalpvvlenarilkncvdakmteedkenalsvldkiytsplcggggsggggsggggsvkmaetcpifydvffavangnellldlsltkvnatepertamkkiqdcyvenglisrvldglvmttissskdcmgeavqntvedlklntlgr

3xKcoil: pI – 9.7; Mw - 2.5 kDa

Feld1: pI – 4.57; Mw – 19.2 kDa.

**A**
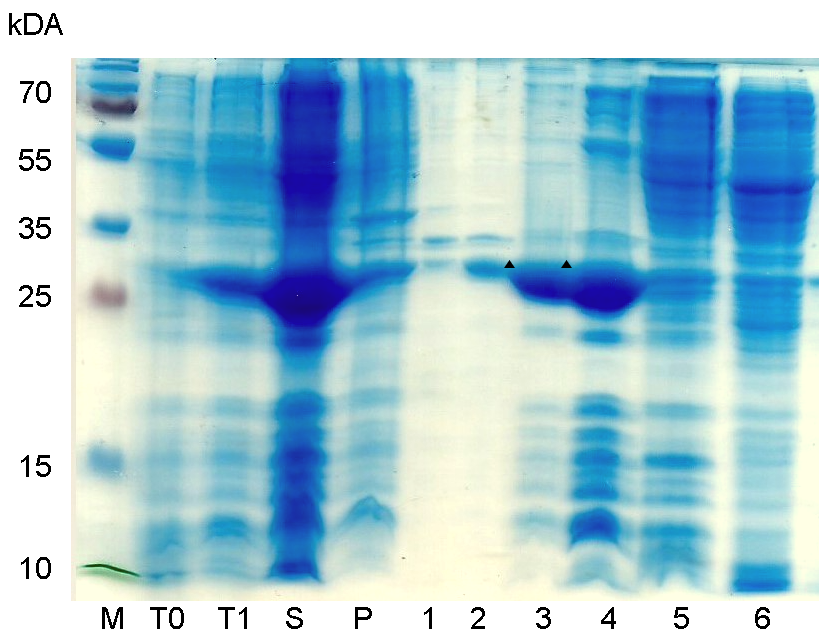
 **B
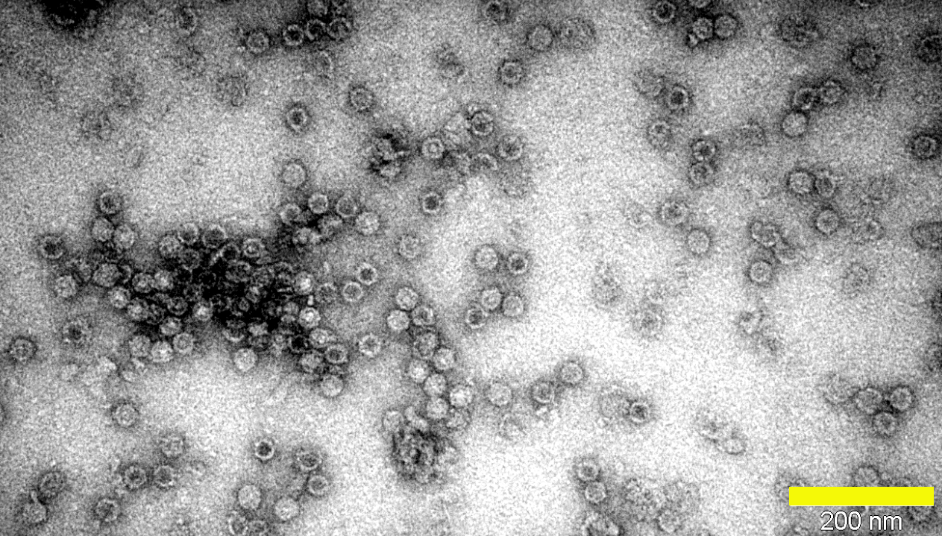
**

**C**
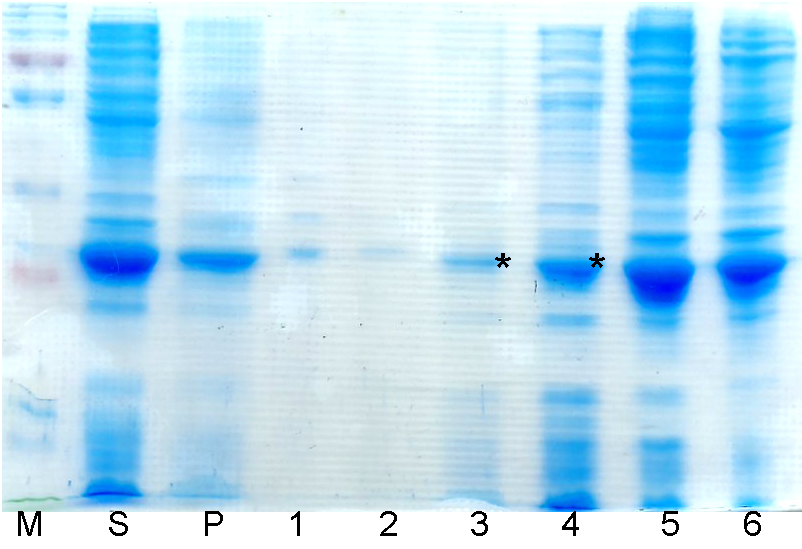
 **D**
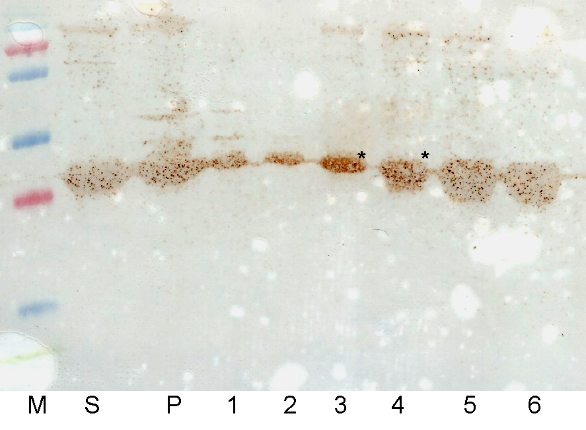
 **E**
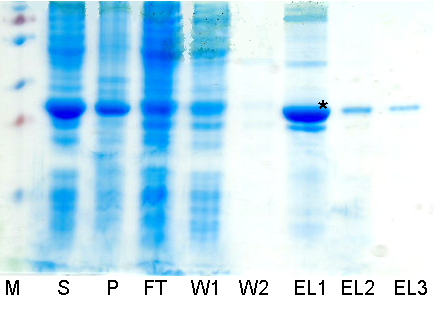


**Suppl. Fig. 8.** Expression and purification of individual EMV-CG4S-SZ18, SZ17-Feld1 and SZ17-Feld1-C6H proteins. A – Coomasie stained SDS – PAGE gel of sucrose gradient of EMV-CG4S-SZ18 protein; B – Electron micrographs of negatively stained VLPs of EMV-CG4S-SZ18. Scale bar 200 nm. C – Coomasie stained SDS – PAGE gel of sucrose gradient of SZ17-Feld1 protein; D – The same samples as (C) analysed in Western blot using anti – Feld1 antibodies; E – Coomasie stained SDS – PAGE gel of SZ17-Feld1-C6H fractions from His-tag column (Protino Ni-IDA 1000 Packed Columns, Macherey-Nagel, Germany).

M - PageRuler Plus Prestained Protein Ladder, 10 to 250 kDa (Thermo Fishers Scientific, USA); T0 - total cell lysate before induction; T1 - total cell lysate after induction; S – soluble proteins in cell lysate; P – insoluble proteins in cell lysate; 1 – 6 – sucrose gradient fractions (60 % - 0 % sucrose); FT – flow- through fraction from His-tag column; W1 – 1^st^ wash of His–tag column; W2 – 2^nd^ wash of His–tag column; EL1 – 1^st^ eluation fraction of His–tag column; EL2 – 2^nd^ eluation fraction from His–tag column; EL3 – 3^rd^ eluation fraction from His–tag column; ▲- triangle denotes the relative position of EMV-SZ18 CP protein; * - asterisk denotes the relative position of SZ17-Feld1 or SZ17-Feld1-C6H protein.

M 1 2 3 4 FT W1 W2 EL1 EL2 EL3

**A**
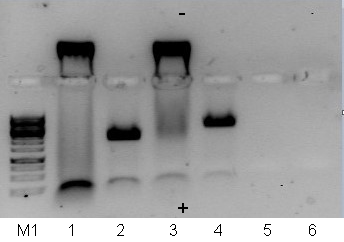
 **B**
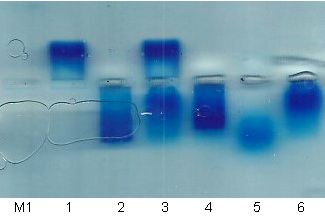


**C
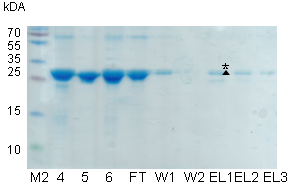
**   **D**
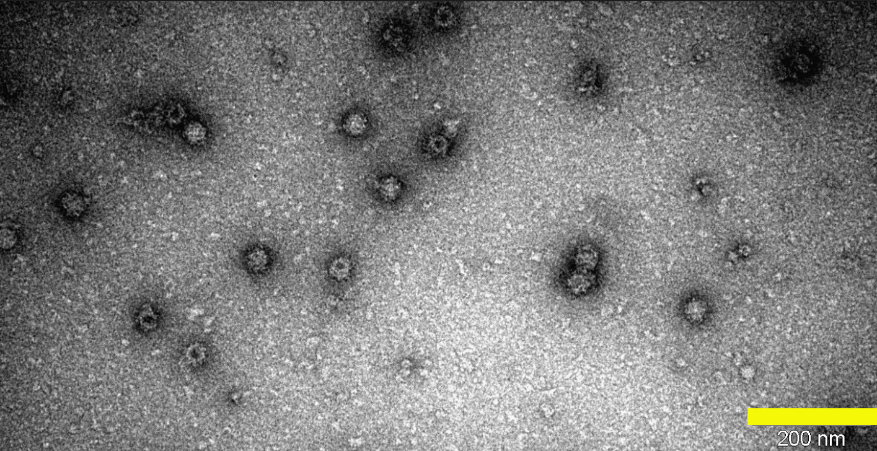


**Suppl. Fig. 9.** Binding analysis of separately expressed EMV-CG4S-SZ18 and SZ17-Feld1-C6H proteins. A - Agarose gel analysis after binding of EMV-SZ18 and Feld1-SZ17-C6H proteins after 1 h incubation at a concentration of 1.5 mg/ml; B – Coomasie stained agarose gel analysis of the same samples as in (A); C - Coomasie stained SDS – PAGE gel of EMV-CG4S-SZ18 + SZ17-Feld1-C6H complex fractions from His-tag column (Protino Ni-IDA 1000 Packed Columns, Macherey-Nagel, Germany); D – Electron micrographs of negatively stained VLPs of dialyzed EL1 fraction of EMV-CG4S-SZ18 CP + SZ17-Feld1-C6H complex after purification on His–tag column. Scale bar 200 nm.

M1 - GeneRuler 1 kb DNA ladder (Thermo Fisher Scientific, USA); M2 - PageRuler Plus Prestained Protein Ladder, 10 to 250 kDa (Thermo Fishers Scientific, USA); 1 –wtEMV VLPs; 2 – EMV-CG4S-SZ18 CP; 3 – wtEMV VLPs + SZ17-Feld1-C6H; 4 – EMV-CG4S-SZ18 + SZ17-Feld1-C6H; 5 – Feld1-C6H; 6 – SZ17-Fedl1-C6H; FT – flow-through fraction from His-tag column; W1 – 1^st^ wash of His – tag column; W2 – 2^nd^ wash of His – tag column; EL1 – 1^st^ eluation fraction of His – tag column; EL2 – 2^nd^ eluation fraction from His – tag column; EL3 – 3^rd^ eluation fraction from His – tag column; (+) and (-) – electrode polarity.

Detailed description of *in vitro* conjugation is found in Materials & Methods (chapter 2.7.). Corresponding proteins of wtEMV, EMV-SZ18 and SZ17-Feld1-C6H were purified and mixed together in a 1:1 ratio according to their concentrations in a volume of 100 µl and incubated for 1 h at room temperature (RT). Additionally, wtEMV conjugation with SZ17-Feld1-CH6 was included as a control experiment.

NAG analysis confirmed wtEMV migration towards negative electrode (Suppl. Fig. 9A; Lane 1) although reduction of positive charge was observed when negatively charged SZ18 sequence was genetically introduced in the wtEMV *CP* (Suppl. Fig. 9A; Lane 2). Opposite effect was achieved for Feld1-C6H protein (Suppl. Fig. 9B; Lane 5) by introducing SZ17 sequence into the *Feld1* gene which slightly reduced its negative charge (Suppl. Fig. 9B; Lane 6). After incubation both samples of wtEMV + SZ17-Feld1-C6H and EMV-SZ18 + SZ17-Feld1-C6H were visualized in agarose gel confirming similar charge for wtEMV + SZ17-Feld1-C6H (Suppl. Fig. 9A; Lane 3) as observed previously for wtEMV although small amount of nucleic acid was bound to positively charged SZ17-Feld1-C6H protein as well. Clearly visible complex formation was indicated for EMV-SZ18 + SZ17-Feld1-C6H (Suppl. Fig. 9A, Lane 4) which slightly shifted towards negative electrode when compared to EMV-SZ18 alone (Suppl. Fig. 9A; Lane 2).

Additionally, to test the binding ability of EMV-SZ18 + SZ17-Feld1-C6H complex we performed purification using His-tag column. We assumed that as EMV-SZ18 + SZ17-Feld1-C6H complex has 6-histidine tag and forms physical binding to EMV-SZ18 the complex the same purification as for 6xhis containing proteins could be performed. We confirmed that both conjugation partners were present in eluated fraction (Suppl. Fig. 9C, EL1) as tymovirus – like particles were observed in TEM. This confirms the high binding ability between separately expressed SZ18/17 partners.

**A**
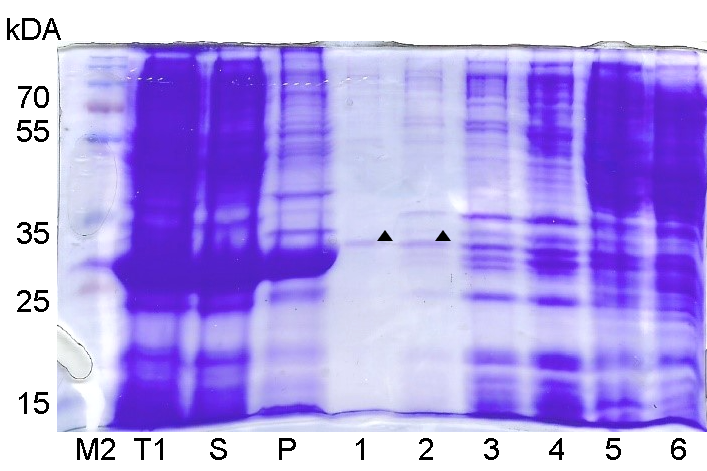
 **B**
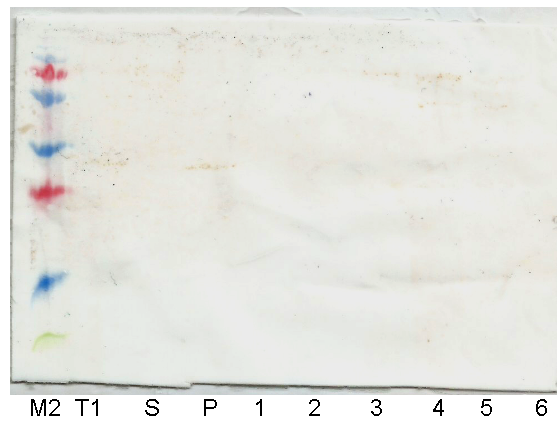
 **C
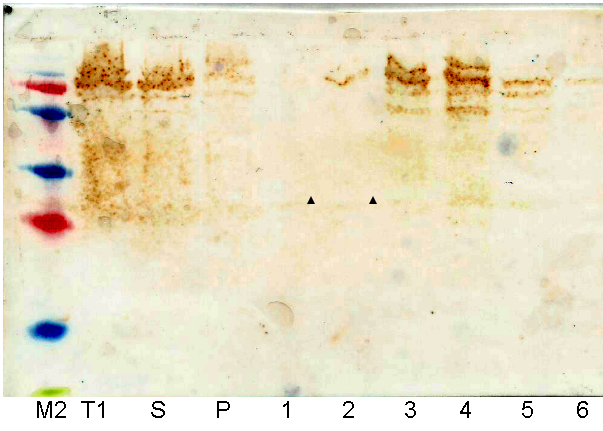
**

**D
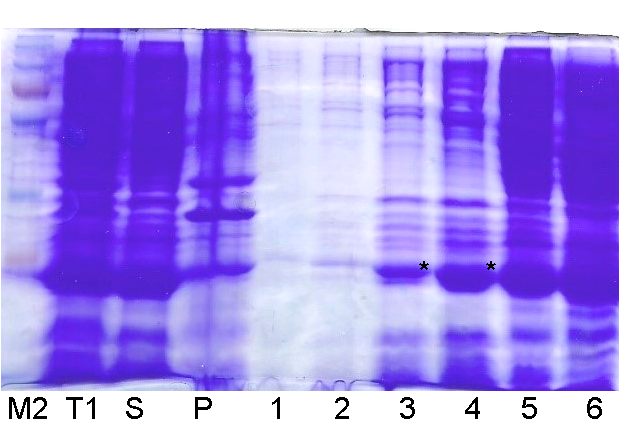
 E
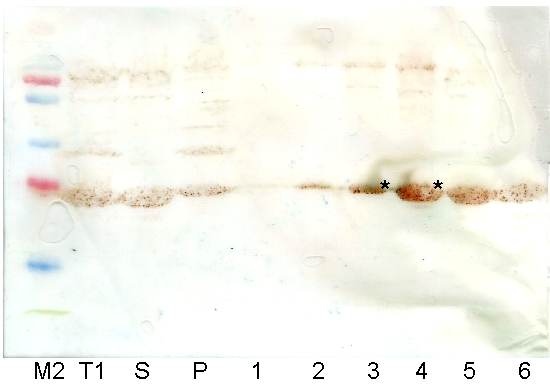
 F
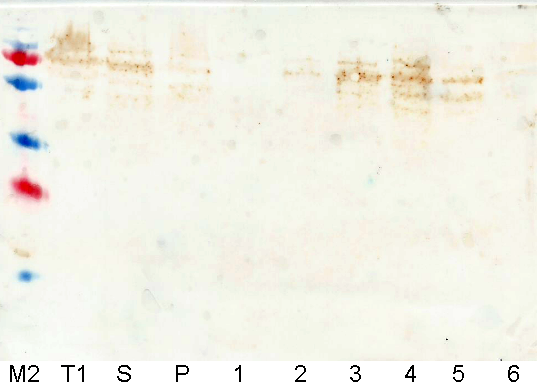
**

**Suppl. Fig. 10.** Separately expressed EMV-CG4S-Ecoil and Kcoil-Feld1 conjugation partner protein purification. A – Coomasie stained SDS – PAGE gel of sucrose gradient of EMV-CG4S-Ecoil CP protein; B – The same samples as (A) analysed in Western blot using anti – Feld1 antibodies; C –– The same samples as (A) analysed in Western blot using anti – EMV antibodies; D – Coomasie stained SDS – PAGE gel of sucrose gradient of Kcoil-Feld1 protein; E - The same samples as (D) analysed in Western blot using anti – Feld1 antibodies; C –– The same samples as (D) analysed in Western blot using anti – EMV antibodies.

M - PageRuler Plus Prestained Protein Ladder, 10 to 250 kDa (Thermo Fishers Scientific, USA); T0 - total cell lysate before induction; T1 - total cell lysate of expression clones after induction; S – soluble protein in cell lysate; P – insoluble proteins in cell lysate; 1 – 6 – sucrose gradient fractions (60 % - 0 % sucrose); ▲- triangle denotes the relative position of EMV-CG4S-Ecoil CP protein; * - asterisk denotes the relative position of Kcoil-Feld1 protein.

**A
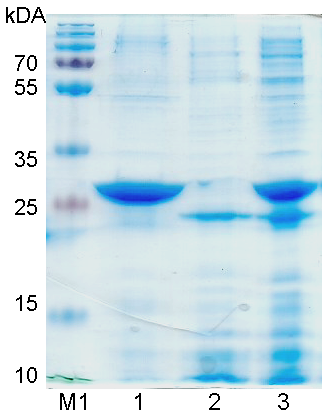
 B
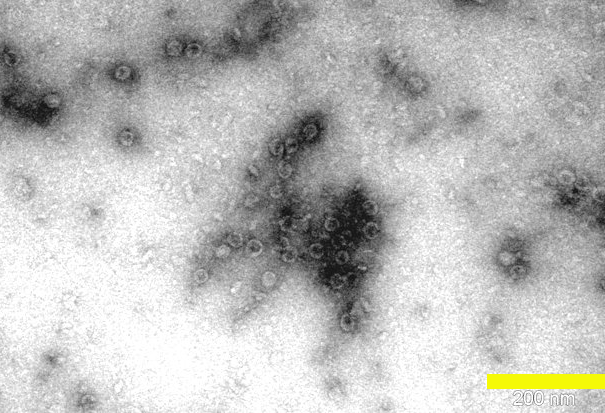
**

**C
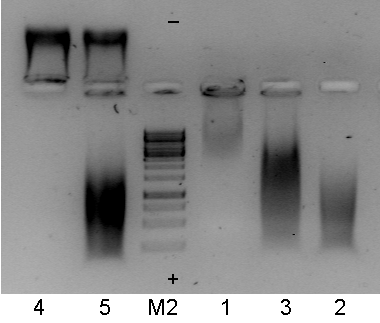
 D
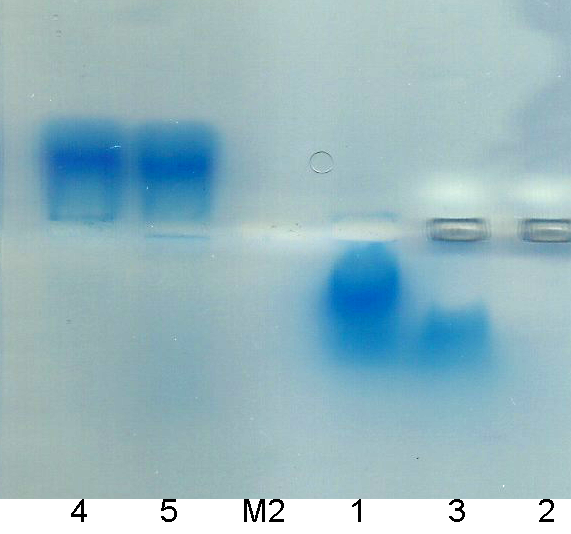
**

**Suppl. Fig. 11.** Analysis of separately expressed EMV-CG4S-Ecoil and Feld1-Kcoil conjugation process. A – Coomasie stained SDS – PAGE gel of EMV-CG4S-Ecoil + Feld1-Kcoil complex samples of 1 mg/ml; B - Electron micrographs of negatively stained VLPs of EMV-CG4S-Ecoil. Scale bar 200 nm; C - Agarose gel analysis of EMV-CG4S-Ecoil and Kcoil-Feld1 purified samples separately and after incubation for 1 h at a concentration of 1 mg/ml; D - Coomasie stained agarose gel analysis of the same samples as in (C).

M1 - PageRuler Plus Prestained Protein Ladder, 10 to 250 kDa (Thermo Fishers Scientific, USA); M2 - GeneRuler 1 kb DNA ladder (Thermo Fisher Scientific, USA); 1 – EMV-CG4S-Ecoil CP; 2 – Kcoil-Feld1; 3 - EMV-CG4S-Ecoil CP + Kcoil-Feld1 using Et-SH for denaturation; 4 – wtEMV CP; 5 – wtEMV CP+ Kcoil-Feld1; (+) and (-) – electrode polarity.

Detailed description of *in vitro* conjugation is found in Materials & Methods (chapter 2.7.). Corresponding proteins of wtEMV, EMV-CG4S-Ecoil (Suppl. Fig. 11B) and Kcoil-Feld1 were purified and mixed together in a 1:1 ratio according to their concentrations in a volume of 100 µl and incubated for 1 h at room temperature (RT) (Suppl. Fig. 11A). Additionally, wtEMV conjugation with Kcoil-Feld1 was included as a control experiment.

NAG analysis confirmed wtEMV migration towards negative electrode (Suppl. Fig. 11C,D; Lane 4) although reduction of positive charge was observed when negatively charged Ecoil sequence was genetically introduced in the wtEMV *CP* (Suppl. Fig. 11C,D; Lane 1). Feld1 protein with introduced Kcoil sequence possess a negative charge (Suppl. Fig.11C,D; Lane 2). After incubation both samples of wtEMV + Kcoil-Feld1 and EMV-Ecoil + Kcoil-Feld1 were visualized in NAG and we confirmed that no physical binding between both proteins was achieved as two distinct bands of nucleic acid was observed (Suppl. Fig. 11C; Lane 5). Clearly visible complex formation was indicated for EMV-Ecoil + Kcoil-Feld1 (Suppl. Fig. 11C,D, Lane 3) which slightly shifted towards positive electrode when compared to EMV-Ecoil alone (Suppl. Fig. 11C; Lane 1).

**
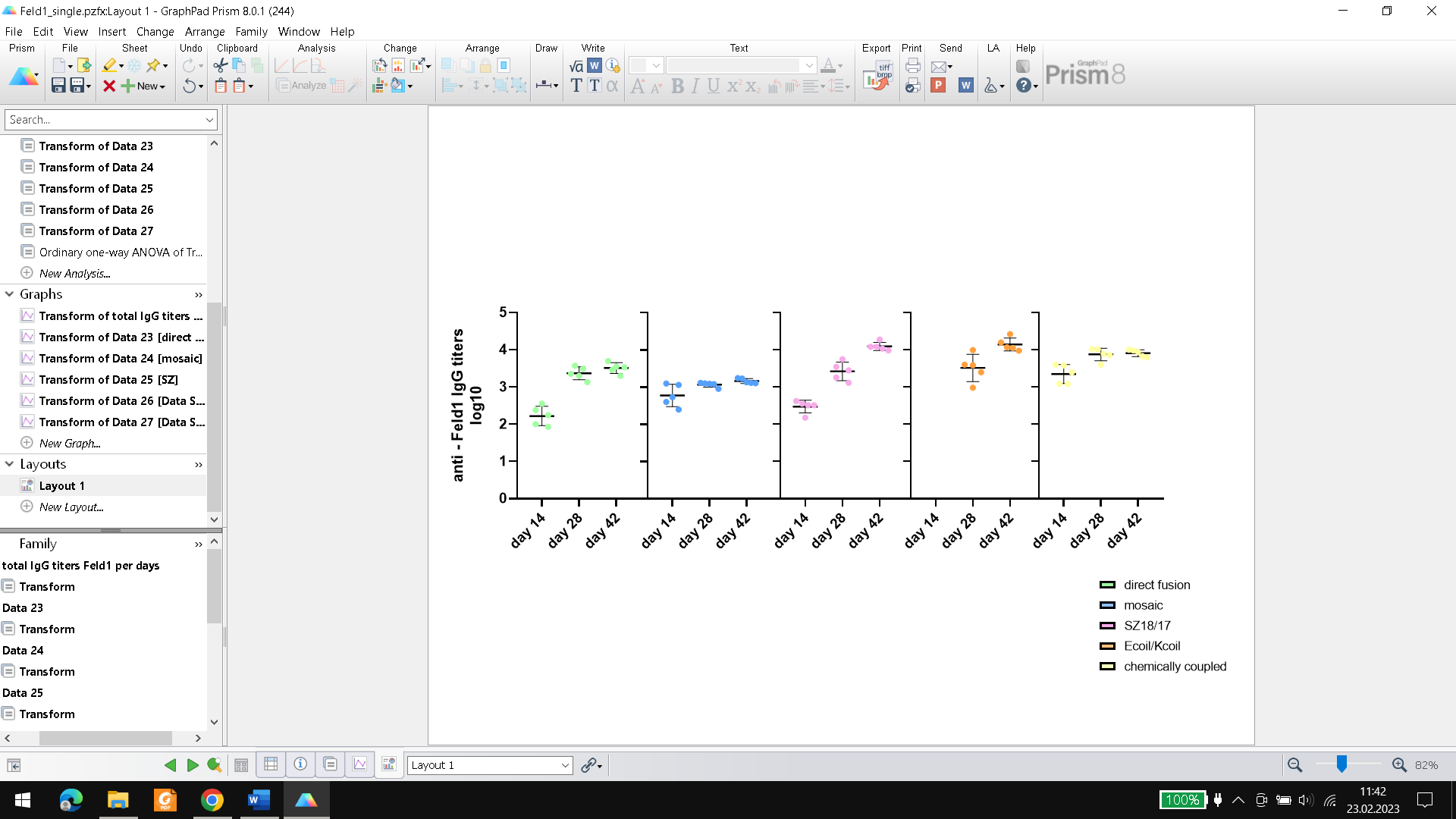
A**
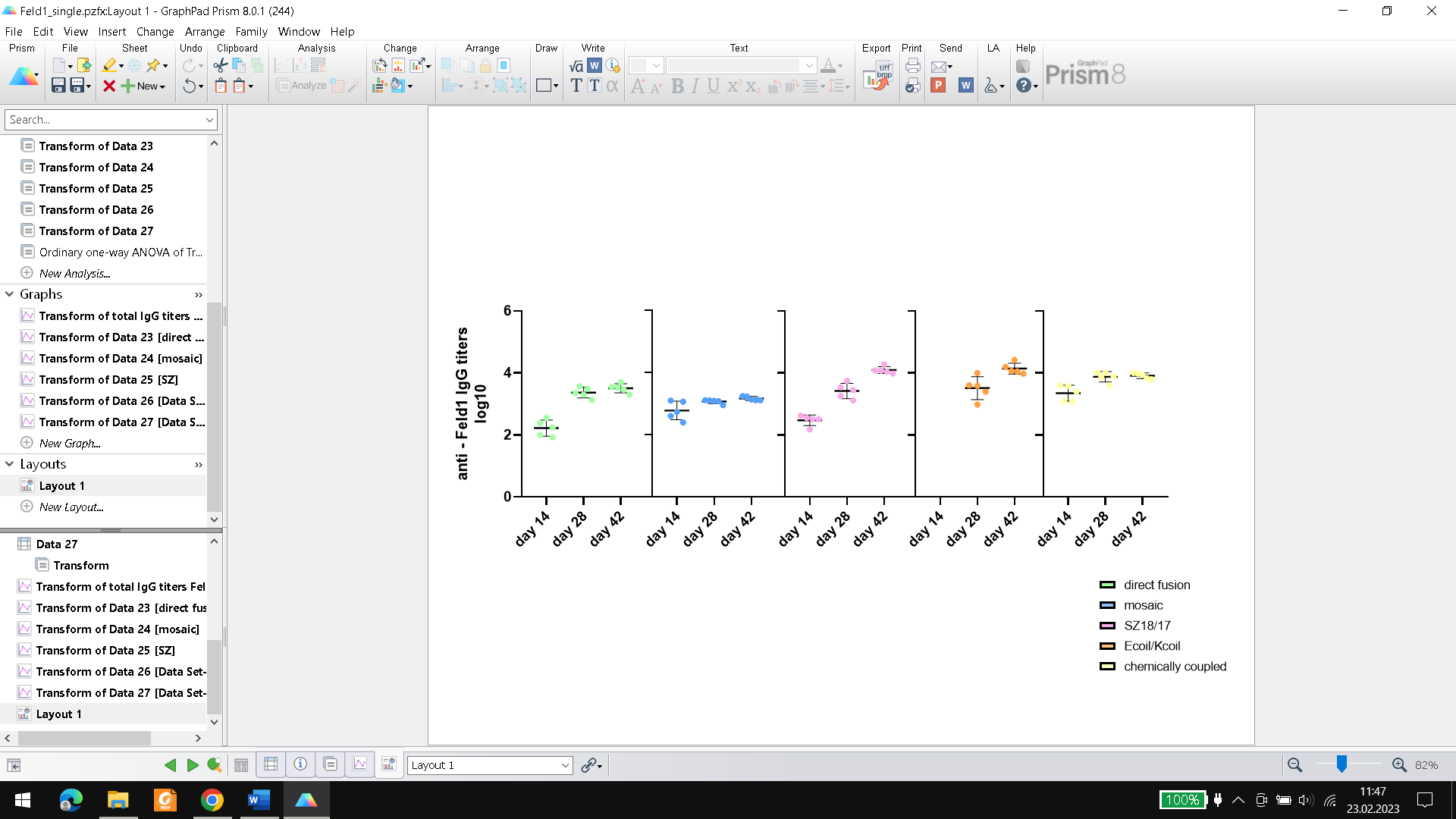


**
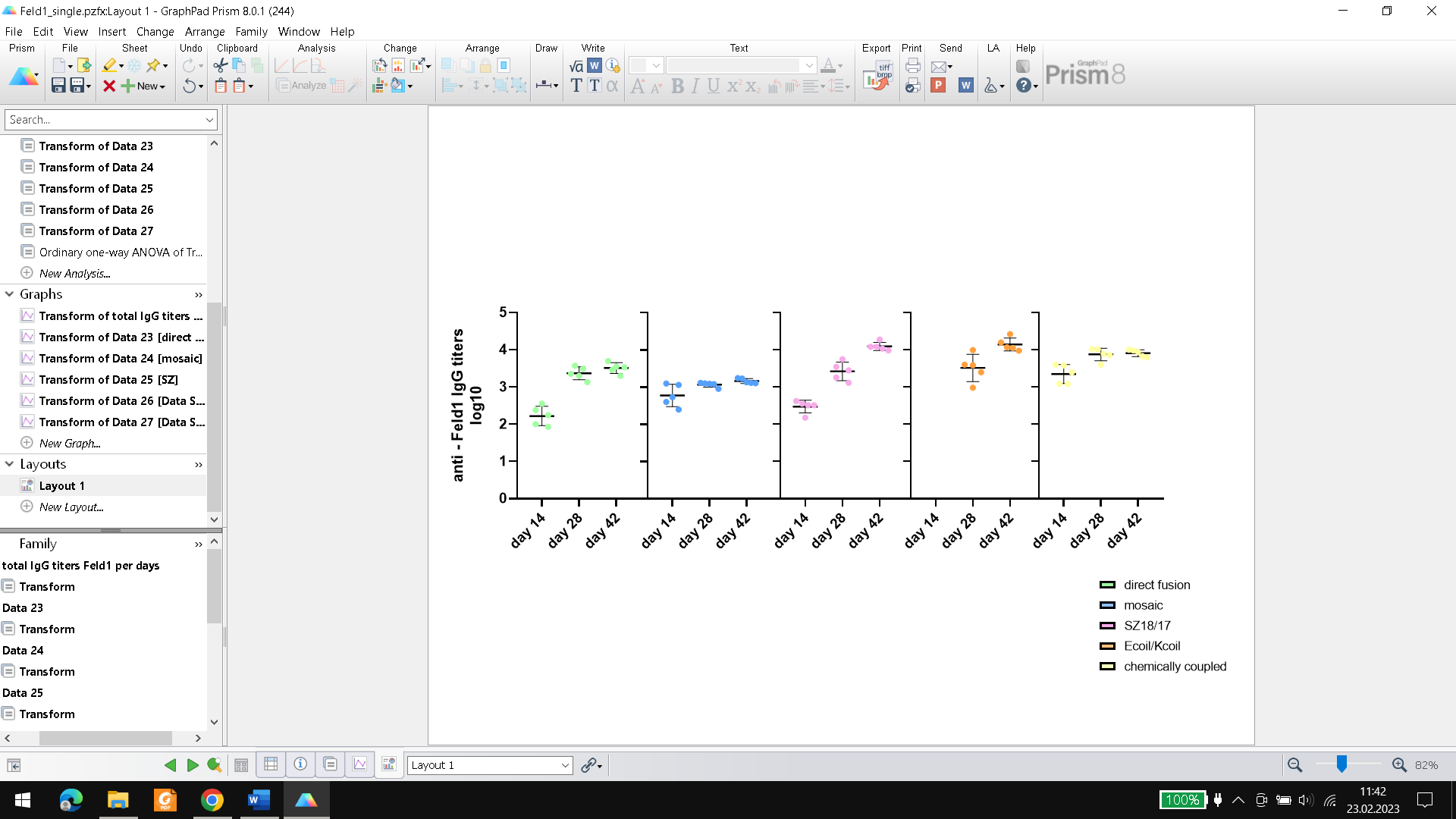
B**
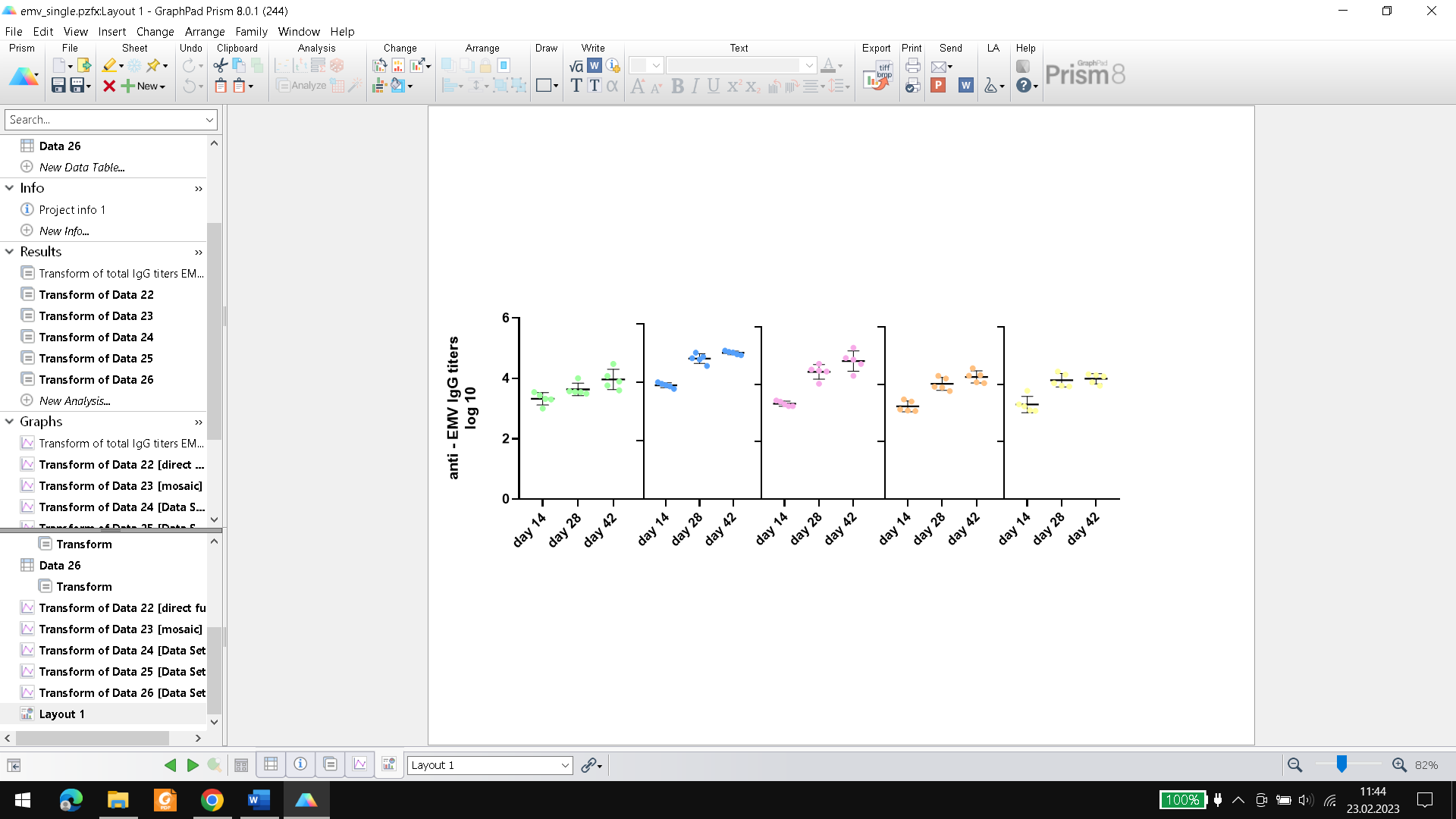


**Suppl. Fig. 12.** IgG titer analysis after vaccination with EMV – Feld1 vaccine variants. A – Log_10_ values (mean ± SEM, n=5) of Feld 1 specific IgG titers on Day 42 for the groups immunized with EMV – Feld1 variants measured at OD 492 nm; B – Log_10_ values (mean ± SEM, n=5) of EMV CP – specific IgG titers on Day 42 for the groups immunized with EMV – Feld1 variants measured at OD 492 nm. Vaccine Groups n = 5.

**
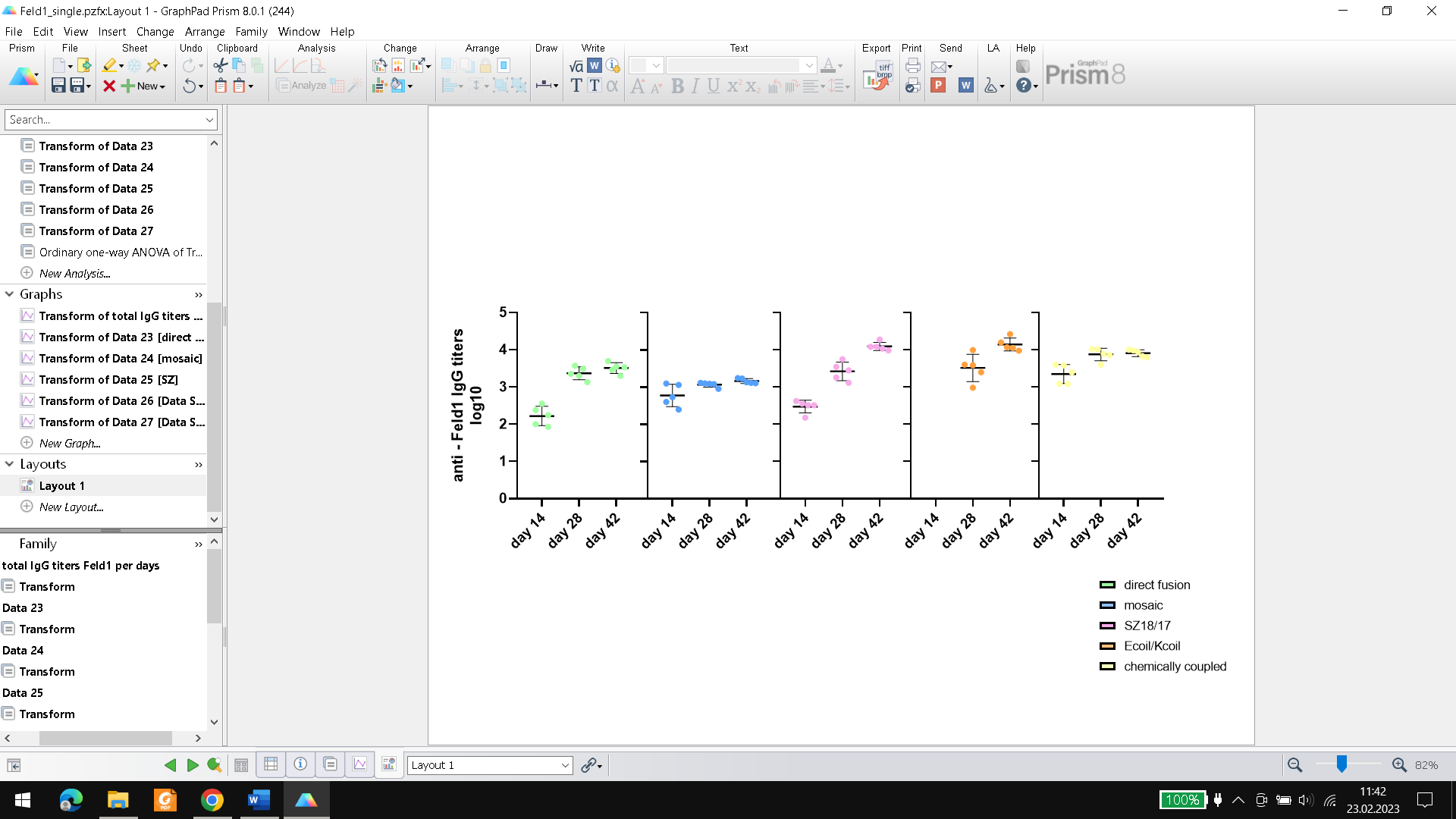
**
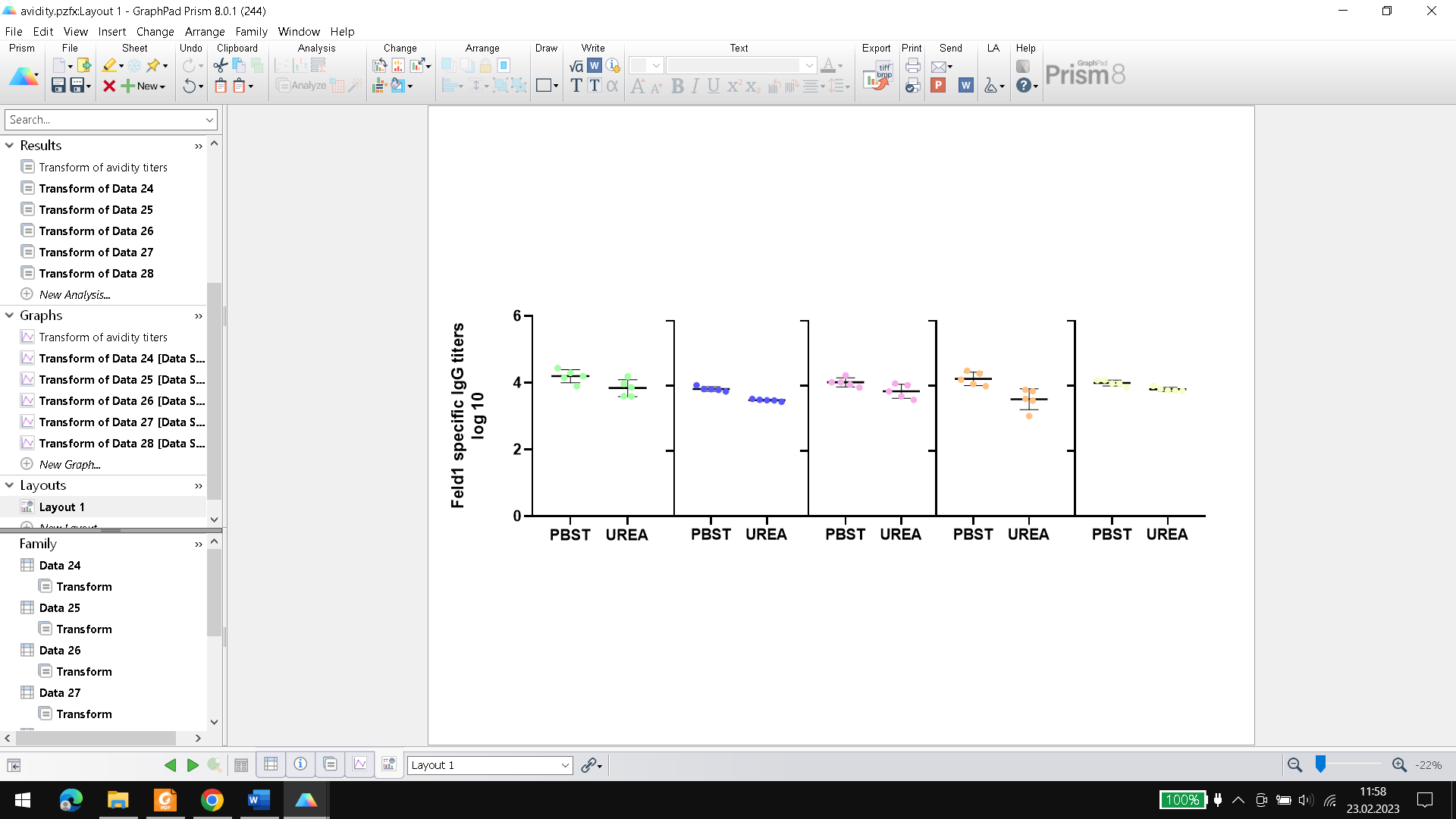


**Suppl. Fig. 13.** Avidity antibody detection after vaccination with EMV – Feld1 vaccine variants. Log_10_ values (mean ± SEM, n=5) of Feld1 specific IgG titers on Day 42 for the groups vaccinated with EMV – Feld1 vaccine variants. After serum incubation one plate was treated with PBS with 0.05% Tween 80 and the other plate with 7 M UREA in PBS with 0.05% Tween 80. Vaccine Groups n = 5.

**
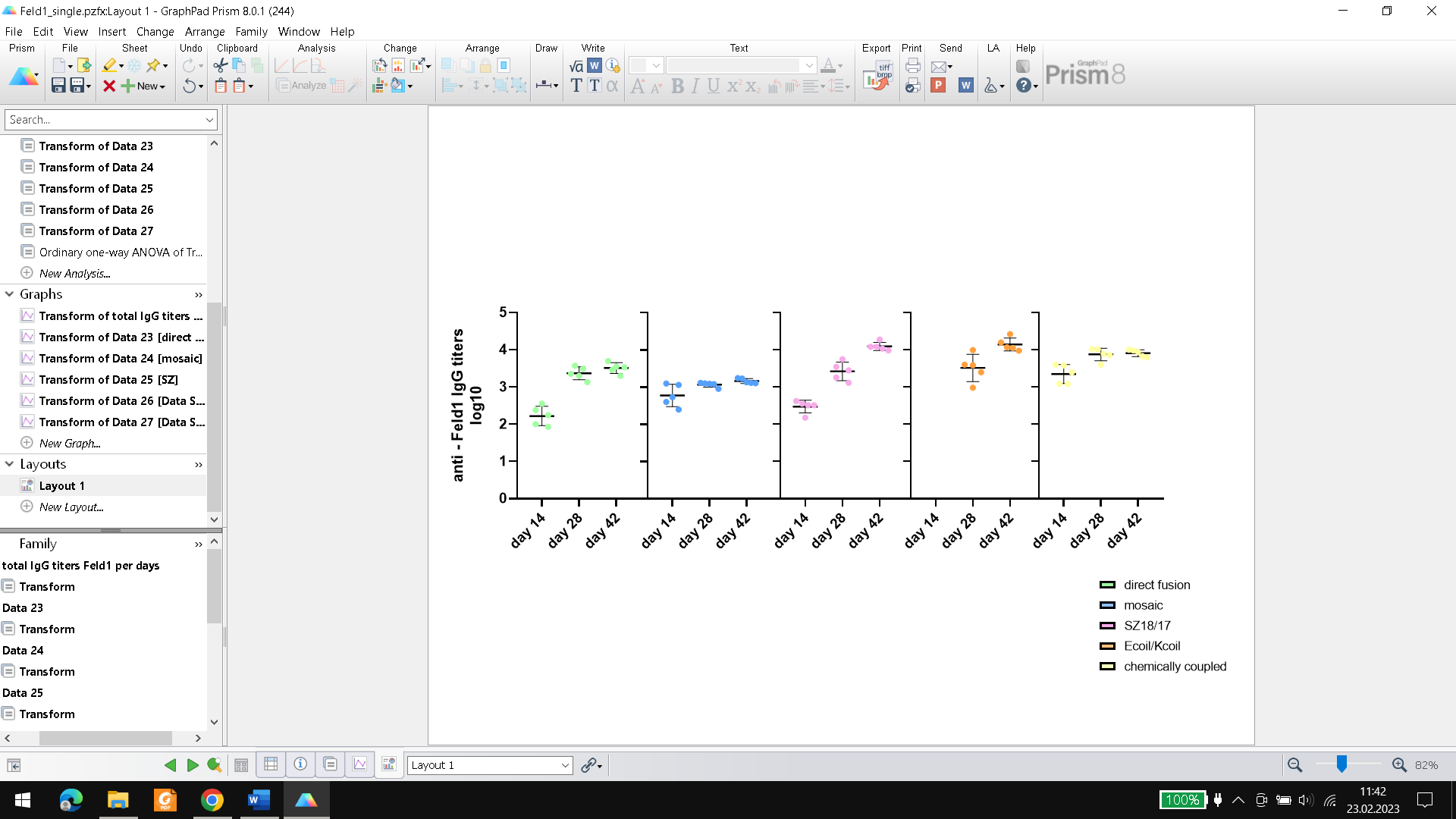
**
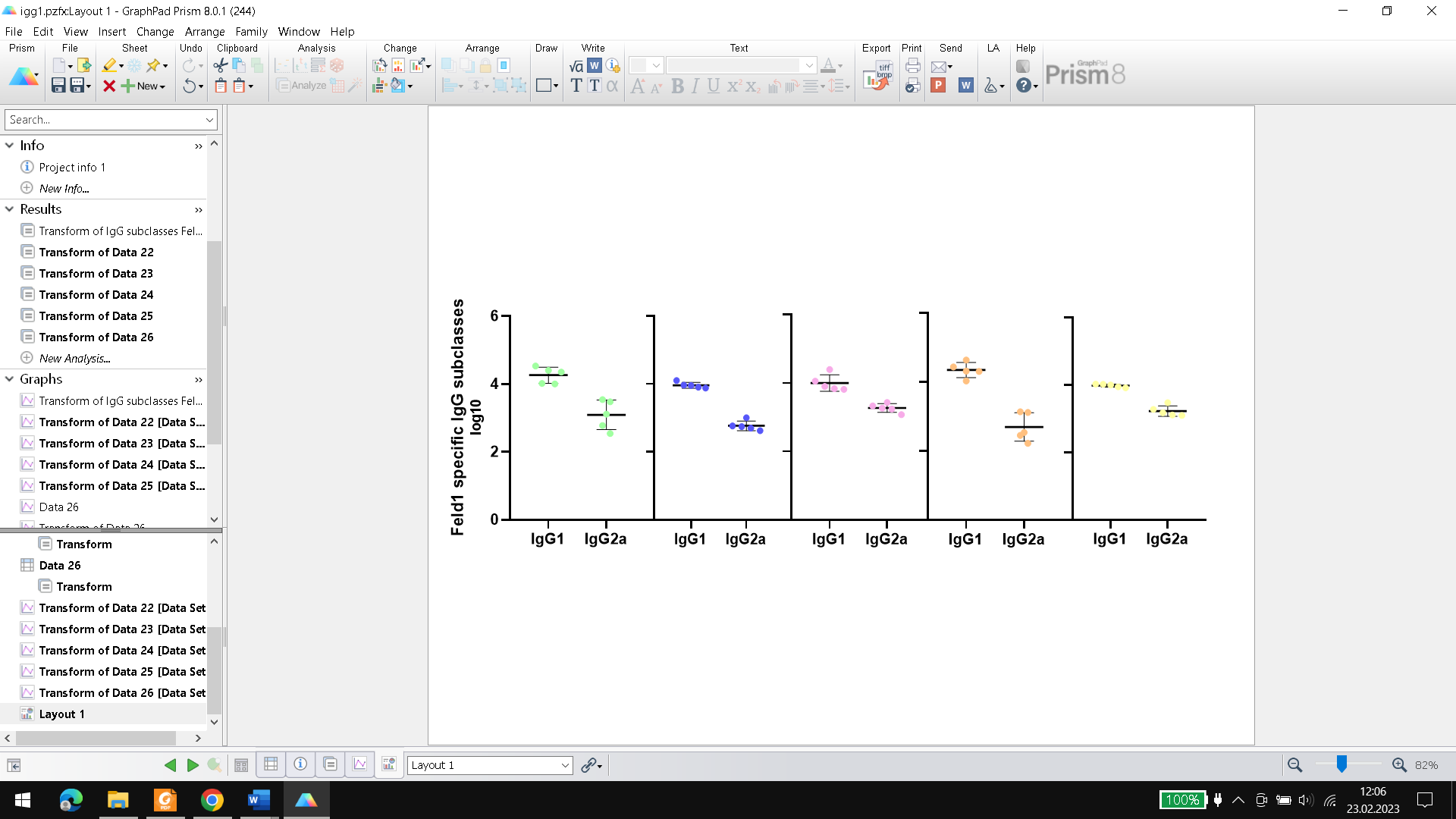


**Suppl. Fig. 14.** Subclass specific antibody detection. Log_10_ values (mean ± SEM, n=5) of Feld 1 specific IgG1 and IgG2a titers measured in Day 42 sera. Vaccine Groups n = 5. ELISA plates were coated with EMV – Feld1 variants for IgG subclass detection in mice sera vaccinated with EMV – Feld1 vaccine variants.
